# Sedation vs. general anaesthesia in patients with atrial fibrillation undergoing catheter ablation: a systematic review and meta-analysis

**DOI:** 10.1093/europace/euaf156

**Published:** 2025-09-18

**Authors:** Beatriz Araújo, André Rivera, Vanessa de Oliveira Tapioca, Lucas M Barbosa, Lucas Caetano, Samuel Navarro Abreu, Sanghamitra Mohanty, Caique M P Ternes, Frans Serpa, Kamala P Tamirisa, André d’Avila, Andrea Natale

**Affiliations:** Departament of Medicine, Nove de Julho University, São Bernardo do Campo, Brazil; Departament of Medicine, Nove de Julho University, São Bernardo do Campo, Brazil; Department of Medicine, Bahiana School of Medicine and Public Health, Salvador, Brazil; Department of Medicine, Federal University of Minas Gerais, Belo Horizonte, Brazil; Department of Medicine, Federal University of Paraíba, João Pessoa, Brazil; Ambulatory Surgery Unit, Policlínica Universitária Piquet Carneiro, Universidade Estadual do Rio de Janeiro, Rio de Janeiro, Brazil; Texas Cardiac Arrhythmia Institute, St. David's Medical Center, 3000 North I-35, Suite 720, Austin, TX 78705, USA; Department of Medicine, Division of Cardiology, Baylor College of Medicine, Houston, TX, USA; Cardiovascular Research Institute, Baylor College of Medicine, Houston, TX, USA; Division of Internal Medicine, University of Texas Southwestern Medical Center, Dallas, TX, USA; Texas Cardiac Arrhythmia Institute, St. David's Medical Center, 3000 North I-35, Suite 720, Austin, TX 78705, USA; Harvard Thorndike Electrophysiology Institute, Beth Israel Deaconess Medical Center, Boston, MA, USA; Texas Cardiac Arrhythmia Institute, St. David's Medical Center, 3000 North I-35, Suite 720, Austin, TX 78705, USA; Department of Biomedicine and Prevention, Division of Cardiology, University of Tor Vergata, Via Montpellier 1, 00133 Rome, Italy; Case Western Reserve University, MetroHealth System, 10900 Euclid Ave, Cleveland, OH 44106, USA

**Keywords:** Atrial fibrillation, Atrial tachyarrhythmia, Catheter ablation, General anaesthesia, Meta-analysis, Sedation

## Abstract

**Aims:**

Catheter ablation is the standard treatment for symptomatic atrial fibrillation (AF) and can be performed under general anaesthesia (GA) or varying levels of sedation to optimize patient comfort and lesion formation. However, the effect of different anaesthesia strategies on AF recurrence rates remains uncertain.

**Methods and results:**

We systematically searched PubMed, Embase, Cochrane, and ClinicalTrials.gov for randomized controlled trials (RCTs) and observational studies comparing outcomes of catheter ablation under GA vs. sedation (including deep, moderate, and conscious sedation). We pooled risk ratios (RR) with 95% confidence intervals (CI) with a random effects model. R version 4.4.1 was used for statistical analyses. Our systematic review and meta-analysis included 6 RCTs and 17 observational studies, corresponding to 12 302 patients assigned to either sedation (*n* = 8952) or GA (*n* = 3350). There was no difference in recurrence of atrial tachyarrhythmias (ATAs) between groups (RR 1.15; 95% CI 0.97–1.36; *P* = 0.10; 95% prediction interval 0.66–2.01). There was no significant subgroup interaction in the recurrence of AF according to sedation type (conscious vs. mild vs. moderate sedation vs. deep sedation) (*P* = 0.20) or AF type (persistent AF vs. non-persistent) (*P* = 0.20).

**Conclusion:**

In patients undergoing catheter ablation for AF, there was no significant difference in recurrence of ATA between GA and sedation.

## Graphical Abstract

**Graphical Abstract:**
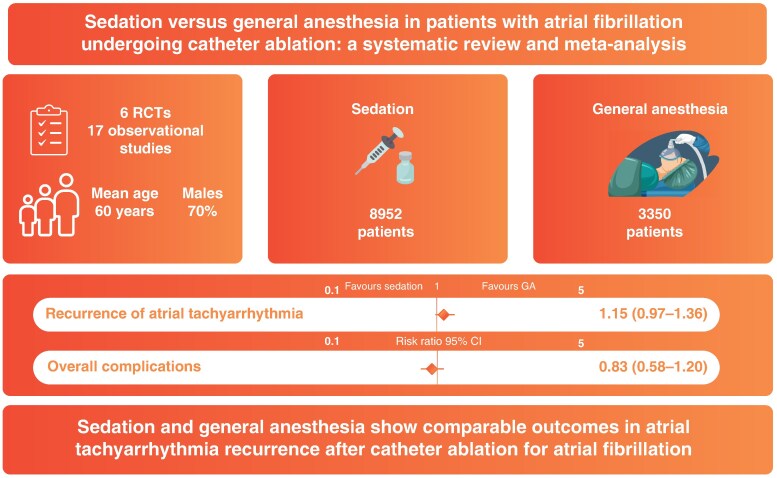

What’s new?

- This is the largest meta-analysis to date directly comparing general anaesthesia (GA) vs. sedation for atrial fibrillation ablation, including over 12 000 patients.
- No significant difference was found in atrial tachyarrhythmia (ATA) recurrence between groups [risk ratio (RR) 1.15; 95% confidence interval (CI) 0.97–1.36; *P* = 0.10].
- A sensitivity analysis restricted to randomized controlled trials showed higher ATA recurrence with sedation (RR 1.76; 95% CI 1.01–3.08; *P* = 0.047).
- There were no significant differences in overall complications or anaesthesia-related complications between groups.
- Secondary endpoints, including procedure time, ablation time, fluoroscopy time, lab occupancy time, and need for re-ablation, were comparable between groups.

## Introduction

Atrial fibrillation (AF) significantly increases the risk of stroke, heart failure, mortality, and recurrent hospital admissions.^1^ Catheter ablation (CA) has demonstrated superior efficacy in achieving sinus rhythm and reducing AF burden compared with medical therapy in patients with AF.^2^

This procedure can be performed either under general anaesthesia (GA) or under local anaesthesia with sedation. The choice of anaesthesia for AF ablation differs among centres and countries, influenced by factors such as available resources, patient characteristics, and the operator’s preference, and it can be stratified as conscious, moderate, or deep.^3^ Currently, there are no established guidelines regarding the impact of the selection of anaesthesia for CA,^4,5^ and current evidence comparing different methods is limited and has so far been conflicting.^6^

Previous meta-analyses have suggested comparable safety and effectiveness between GA and sedation,^6,7^ but multiple recent studies have reported variable findings regarding procedural outcomes.^8,9^ While prior research has primarily focused on qualitative comparisons, the precise magnitude of GA’s impact on efficacy and safety endpoints remains unclear. To address this gap, we performed an updated meta-analysis to quantitatively assess the effect size of GA vs. sedation during AF ablation.

## Methods

The systematic review and meta-analysis were performed and reported following the Cochrane Collaboration Handbook for Systematic Reviews of Interventions and the Preferred Reporting Items for Systematic Reviews and Meta-Analysis (PRISMA) Statement guidelines (see Supplementary material online, *Methods S1* and *S2*).^10,11^ The prospective meta-analysis protocol was registered at the International Prospective Register of Systematic Reviews (PROSPERO; CRD42024589329) in September 2024.

### Data source and search strategy

We systematically searched PubMed, Embase, Cochrane, and ClinicalTrials.gov from inception until our last search in June 2025 using the terms ‘catheter ablation’, ‘pulmonary vein isolation’, ‘sedation’, and ‘general anesthesia’ to identify studies comparing outcomes of GA vs. sedation. There was no restriction concerning the publication date or language. Two authors (B.A. and V.d.O.T.) independently screened titles and abstracts and evaluated the articles in full for eligibility based on pre-specified criteria. Discrepancies were resolved in a panel discussion between authors. The complete search strategy is given in Supplementary material online, *Methods S3*.

### Eligibility criteria

We considered studies eligible for inclusion if they (i) were randomized controlled trials (RCTs) or observational studies; (ii) compared GA with sedation, including conscious, deep, mild, or moderate sedation; and (iii) provided data on clinical and peri-procedural endpoints. We excluded studies comparing only different sedation strategies and conference abstracts.

### Data extraction

Three authors (B.A., L.M.B., and V.d.O.T.) independently extracted the data for each study using a standardized study form to determine authors, study publication year, energy source used, sample size, follow-up period, endpoint definition, baseline patient characteristics, procedure characteristics, and drugs used during sedation and GA. Any discrepancies were settled through a panel discussion with a fourth author (A.R.). Drugs used in sedation and GA were described in Supplementary material online, *Methods S4*.

### Endpoints

The primary endpoint was the (i) recurrence of atrial tachyarrhythmias (ATA). Secondary endpoints included (ii) procedural time, (iii) ablation time, (iv) lab occupancy time, (v) fluoroscopy time, (vi) need for redo ablation, (vii) overall complication rates, and (viii) anaesthesia-related complications. Across studies, ATA was predominantly defined as a composite endpoint of ATA, atrial tachycardia, and atrial flutter. All-cause mortality was not assessed given the limited number of studies reporting this endpoint. Detailed definitions for ATA recurrence, procedural time, and overall complications are provided in Supplementary material online, *Methods S5* and *S6*.

### Subgroup, sensitivity, and meta-regression analyses

We conducted pre-specified subgroup analyses for the primary outcome. Studies were grouped based on (i) sedation strategy, (ii) AF type (paroxysmal vs. persistent AF), (iii) study design (RCTs and propensity score matched studies vs. studies without adjustment), (iv) energy source used [radiofrequency ablation (RFA) vs. cryoballoon ablation (CBA) vs. pulsed field ablation (PFA)], (v) study continent, and (vi) ablation strategy [pulmonary vein isolation (PVI) only vs. PVI plus additional ablation]. We also performed a meta-analysis restricted to RCTs. Regarding complications, subgroup analyses were performed based on the energy source and ablation strategy (PVI only vs. PVI plus additional ablation). Leave-one-out sensitivity was performed to ensure the results were not dependent on a single study.

Additionally, univariate meta-regression analyses were conducted to investigate potential associations between ATA recurrence and follow-up duration, year of publication, proportion of males, mean age, and hypertension. We performed a sensitivity analysis of studies with follow-up longer than 2 years to assess the durability of GA’s effects on AF recurrence over an extended period.

### Quality assessment

Two independent authors (L.C. and V.d.O.T.) assessed the risk of bias of recurrence of ATA in the included non-randomized studies using the Cochrane tool for assessing the Risk of Bias in Non-Randomized Studies of Interventions (ROBINS-I).^12^ For RCTs, we used Cochrane’s Collaboration tool for assessing the risk of bias in randomized trials (RoB 2).^13^ Any disagreements were resolved through consensus between authors. We explored the potential for publication bias by visual inspection of the comparison-adjusted funnel plots and Egger’s regression test for the primary endpoint.^14^

### Statistical analysis

We used the Mantel–Haenszel random effects model for all endpoints. We employed risk ratios (RRs) and 95% confidence intervals (CIs) as the measure of effect size for binary endpoints. We calculated 95% prediction intervals to reflect the expected range of effects in future studies. For continuous endpoints, we utilized weighted mean differences (MDs) with 95% CIs. A restricted maximum likelihood estimator was used to calculate heterogeneity variance *t*^2^.

We assessed heterogeneity using Cochran’s Q statistic and Higgins and Thompson’s *I*² statistic, which estimates the proportion of variability due to between-study heterogeneity. We interpreted *I*² values of 0%, ≤25%, ≤50%, and >50% as indicating no observed, low, moderate, and substantial heterogeneity, respectively. All tests were two-tailed, and *P* < 0.05 was considered significant. If necessary, means and standard deviations were estimated.^15^ We used R version 4.4.1 for all calculations and graphics.^16^

## Results

### Study selection and baseline characteristics

**Figure 1:**
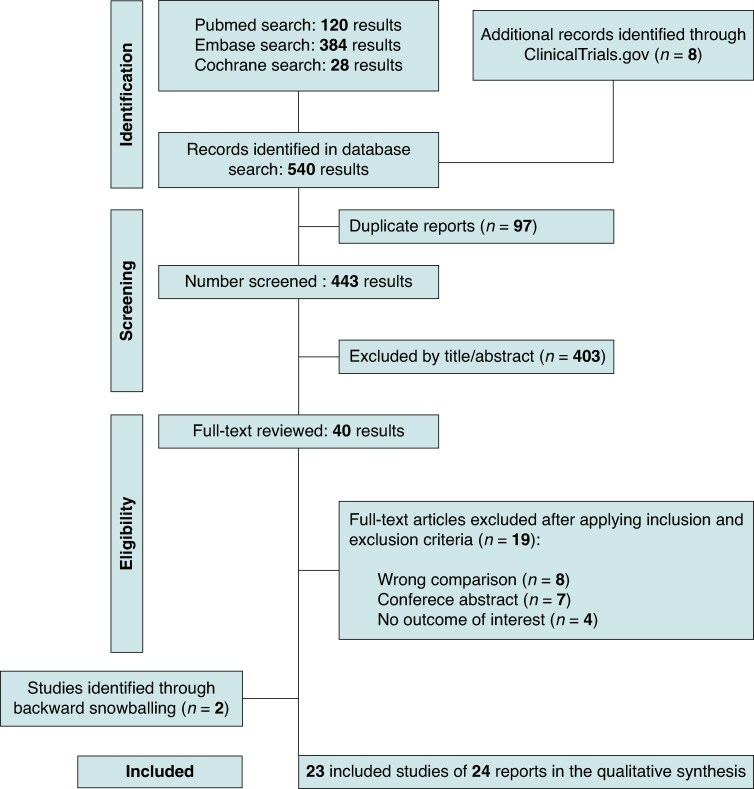
PRISMA flow diagram of study screening and selection.

Our systematic search yielded 446 potential articles. After deduplication and initial title and abstract screening, 36 full-text articles were retrieved in full for possible inclusion. Ultimately, 24 reports from 23 studies met all inclusion criteria and were included in the primary analysis, five of which were RCTs.^8,9,17–38^ Comprehensive details of the study selection are detailed in *Figure 1*.

We included a total of 12 302 patients, of whom 8952 (72.7%) underwent sedation during AF ablation. The mean age of patients was 63.9 years, and the proportion of males was 70.4% (*Table 1*). Radiofrequency ablation was the most used energy source (82%), and four studies investigated PFA. Among studies reporting AF type, 1434 (57%) were classified as persistent AF in the GA group compared to 3196 (39%) in the sedation group. In the pooled analysis, 666 patients (5.4%) were enrolled from RCTs.

**Table 1:** Baseline characteristics of included studies

Study, year	Study design	Patients, *n*	Left atrial diameter, mm	Hypertension, %	Sedation type	Mean age, y	Male sex, *n*	AF type	Energy source	Follow-up^a^
		Sedation/GA	Sedation/GA	Sedation/GA		Sedation/GA	Sedation/GA			Sedation/GA
**Bun, 2014^17^**	Observational, prospective	45/45	41/43	31/49	Conscious sedation	61/60	33/30	PAF, PsAF	RFA	323/351 days
**Calvert, 2024 (1)^9^**	Observational, retrospective	51/32	NA	29/59	Mild conscious sedation	59/58	31/19	PAF, PsAF	RFA	12 months
**Calvert, 2024 (2)^38^**	Observational, prospective	8/15	NA	25/53	Mild conscious sedation	63/61	7/9	PAF, PsAF	PFA	101 days
**Chikata, 2017^18^**	Observational, retrospective	69/107	39.9/40.8	46/45	Conscious sedation	65/66	54/80	PAF, PsAF	RFA	519/339 days
**Di Biase, 2009^19^**	RCT	25/25	41/42	36/32	Conscious sedation	58/57	76/72	PAF	RFA	12 months
**Di Biase, 2011^20^**	RCT	128/129	40/42	41/42	Conscious sedation	58/60	97/95	PAF	RFA	16/15 months
**Firme, 2012^21^**	RCT	16/16	NA	68/56	Deep sedation	53/55	10/10	PAF, PsAF	RFA	3 months
**Kanthasamy, 2023^22^**	Observational, prospective	35/25	42.5	NA	Conscious sedation	64	43	PAF, PsAF	RFA	3 months
**Kuno, 2023^23^**	Observational, retrospective	13/17	34/36	62/53	Conscious sedation	65/67	9/8	PAF	RFA	NA
**Lo, 2025^24^**	Observational, prospective	56/262	39.9	NA	Conscious sedation	65	7	PsAF, PAF	PFA	5 months
**Mahmoodi, 2023^25^**	Observational, retrospective	182/118	41.5/44.7	43/50	Conscious sedation	63/66	119/77	PsAF	CBA/RFA	12 months
**Martin, 2018^26^**	Observational, retrospective	220/72	NA	35/33	Conscious sedation	59/60	181/57	PsAF	RFA	12 months
**Minciun**ă**, 2024^28^**	Observational, retrospective	84/47	40/41	71/57	Mild conscious sedation	60/58	55/32	PAF, PsAF	RFA	6 months
**Moravec, 2021^27^**	RCT	73/77	41/42	60/62	Conscious sedation	56	54/51	PAF	RFA	12 months
**Riis-Vestergaard, 2024^8^**	Observational, retrospective	6421/1536	NA	48/53	Conscious sedation	62/61	4504/1060	PAF, PsAF	CBA/RFA	5 years
**Rillig, 2024^37^**	Observational, retrospective	40/23	43/42	62/65	Deep sedation	65/68	27/14	PAF, PsAF	PFA	NA
**Sta**š**kov**á**, 2017^30^**	RCT	25/25	43.4/40.4	48/64	Conscious sedation	59	19/14	PAF	RFA	12 months
**Sochorová, 2025^29^**	RCT	85/42	NA	50/24	Conscious/deep sedation	62/64	55/21	Non-PAF	PFA	NA
**Wang, 2021^32^**	Observational, retrospective	203/148	37/36	40/42	Conscious sedation	61/62	121/87	PAF	RFA	12 months
**Wang, 2024^31^**	Observational, prospective	109/36	40.9/40.4	57/20	Conscious sedation	61/62	82/28	PAF	RFA	12 months
**Wasserlauf, 2016^34^**	Observational, retrospective	119/55	39/38	41/54	Moderate sedation	61/62	81/38	PAF	CBA	11 months
**Wasserlauf, 2020^33^**	Observational, prospective	53/47	37/36	41/47	Moderate sedation	65/64	29/27	PAF	CBA	NA
**Xu, 2017^35^**	Observational, retrospective	278/220	38/37	46/42	Conscious sedation	60/60	179/138	PAF, PsAF	RFA	12 months
**Yokokawa, 2022^36^**	Observational, retrospective	534/276	44/45	58/65	Deep sedation	63/64	370/187	PAF, PsAF	RFA	43 months

AF, atrial fibrillation; CBA, cryoballoon ablation; GA, general anaesthesia; LAD, left atrial diameter; NA, not available; PAF, paroxysmal atrial fibrillation; PFA, pulsed field ablation; PsAF, persistent atrial fibrillation; RCT, randomized controlled trial; RFA, radiofrequency ablation.

^a^Mean or median.

### Efficacy endpoints

**Figure 2:**
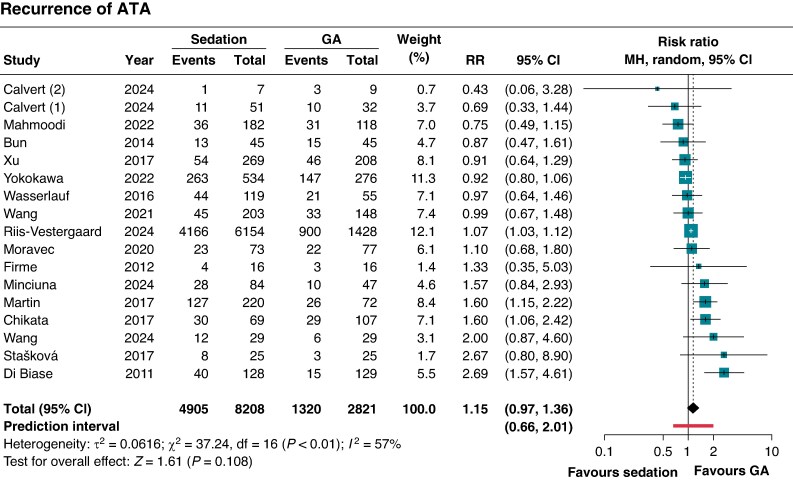
Meta-analysis of recurrence of ATA in patients with AF undergoing CA with sedation vs. GA. Forest plots presenting the (*A*) RR and 95% CI and (*B*) the risk difference and 95% CI for each treatment strategy. ATA, atrial tachyarrhythmia; CA, catheter ablation; CI, confidence interval; MH, Mantel–Haenszel; GA, general anaesthesia; RR, risk ratio.

There was no significant difference in ATA recurrence rates between groups (RR 1.15; 95% CI 0.97–1.36; *P* = 0.10; 95% prediction interval 0.66–2.01; *Figure 2A*).

### Subgroup, sensitivity, and meta-regression analyses

**Figure 3:**
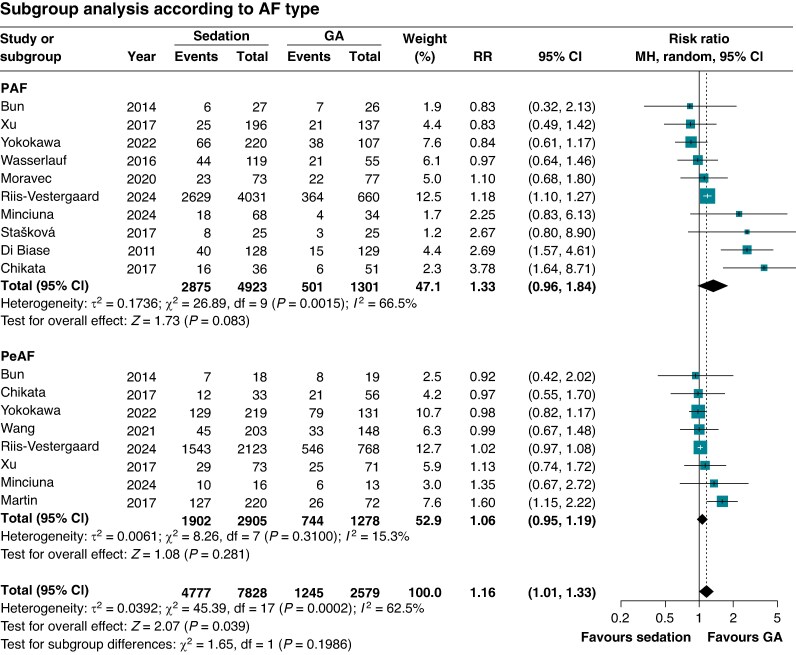
Subgroup analysis of recurrence of ATA showed no significant interaction when stratified by AF type (paroxysmal vs. persistent AF). Forest plots presenting the RR and 95% CI for each treatment strategy according to AF type. ATA, atrial tachyarrhythmia; CI, confidence interval; MH, Mantel–Haenszel; GA, general anaesthesia; RR, risk ratio.

There was no significant subgroup interaction of ATA recurrence based on (i) sedation strategy (*P* = 0.20), (ii AF type (*P* = 0.20; *Figure 3*), (iii) energy source used (*P* = 0.56), (iv) study continent (*P* = 0.40), or (v) ablation strategy (*P* = 0.60). However, a significant subgroup interaction was observed for study design (*P* = 0.03), showing a higher relative risk of recurrence of sedation vs. GA among RCTs and propensity score matched relative to studies without adjustment (RR 1.79; 95% 1.14–2.83 vs. RR 1.05; 95% 0.91–1.21) (see Supplementary material online, *Figure S1*). Leave-one-out sensitivity analysis demonstrated that the results were consistent after each included study was omitted in the recurrence of the ATA endpoint (see Supplementary material online, *Figure S2*).

In a sensitivity analysis restricted to RCTs, there was a higher risk of ATA recurrence with sedation compared to GA (RR 1.76; 95% CI 1.01–3.08; *P* = 0.047). In an analysis restricted to studies with longer follow-up, there was no significant difference between groups (RR 1.28; 95% CI 0.89–1.84; *P* = 0.18; *I*^2^: 79%) (see Supplementary material online, *Figure S3*).

No significant associations were observed between ATA recurrence and follow-up duration (*P* = 0.48), hypertension (*P* = 0.71), year of publication (*P* = 0.06; Supplementary material online, *Figure S4D*), or mean age (*P* = 0.06). However, a significant association was found for proportion of males (*P* < 0.01) (see Supplementary material online, *Figure S4*).

### Safety endpoint

**Figure 4:**
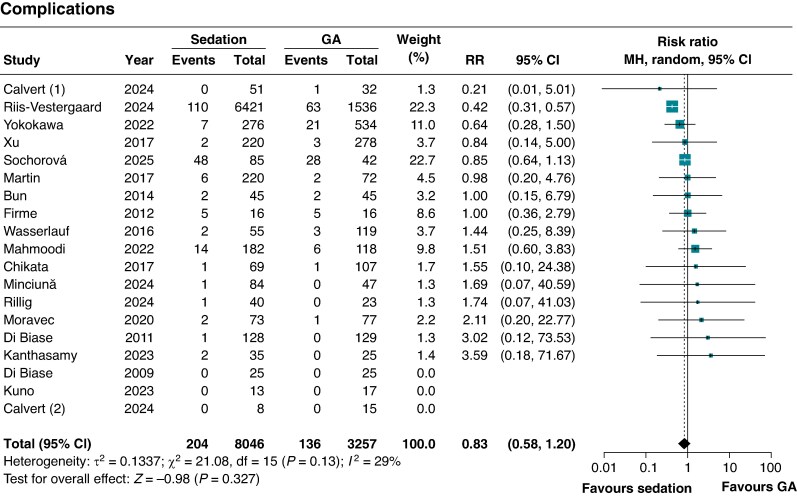
Meta-analysis of the complications in patients with AF undergoing CA with sedation vs. GA. Forest plots presenting the RR and 95% CI for each treatment strategy. ATA, atrial tachyarrhythmia; CI, confidence interval; MH, Mantel–Haenszel; GA, general anaesthesia; RR, risk ratio.

There was no significant difference between groups in overall complications (RR 0.83; 95% CI 0.58–1.20; *P* = 0.33; *I*^2^ = 29%; *Figure 4*). No significant difference was observed in anaesthesia-related complications between the groups (RR 1.49; 95% CI 0.59–3.79; *P* = 0.39; *I*²=0%). There was no significant subgroup interaction according to ablation strategy (*P* = 0.12) and energy source (*P* = 0.80) (see Supplementary material online, *Figure S5*).

### Secondary endpoints

There was no significance difference in procedural duration (MD 2.7 min; 95% CI −12.3–17.7; *P* = 0.72; *I*^2^ = 96%), fluoroscopy time (MD 2.0 min; 95% CI −2.8–6.8; *P* = 0.41; *I*^2^ = 96%), ablation time (MD 0.0 min; 95% CI −3.1–3.1; *P* = 0.99; *I*^2^ = 96%), need for redo ablation (RR 1.38; 95% CI 0.92–2.06; *P* = 0.11; *I*^2^ = 73%; Supplementary material online, *Figure S6D*), or lab occupancy time (MD −8.9 min; 95% CI −23.1–5.3; *P* = 0.20; *I*^2^ = 88%) (see Supplementary material online, *Figure S6*).

### Risk of bias assessment

ROBINS-I identified 11 studies with a serious risk of bias^9,17,18,26,28,8,32,34–36,9^ and two with moderate concerns of bias (see Supplementary material online, *Figure S7A*).^25,31^ Among RCTs, RoB-2 identified all studies as having some concerns of bias, due to the absence of a pre-specified plan and the awareness of patients and caregivers regarding the intervention allocation (see Supplementary material online, *Figure S7B*). Funnel plot analysis and Egger regression test did not detect evidence of publication bias for the primary endpoint (*P* = 0.44; Supplementary material online, *Figure*  *S8*).

## Discussion

This systematic review and meta-analysis of 23 studies including 12 302 patients compared GA vs. sedation during CA for AF. Our main findings were (i) no significant difference in ATA recurrence between groups; (ii) similar overall and anaesthesia-related complication rates; and (iii) no significant differences in ablation, procedural, or fluoroscopy times.

The lack of a significant difference in ATA recurrence suggests that the choice between GA and sedation may not directly affect rhythm outcomes. Although GA theoretically offers advantages such as better control of patient movement, optimized ventilation, and enhanced catheter stability, these benefits did not translate into lower recurrence rates in the overall analysis.^4,23^ Nevertheless, patients undergoing GA were generally more complex, with a higher proportion of patients in the with persistent AF compared to the sedation group (58% vs. 39%) in this pooled population. This imbalance may have underestimated the treatment effect in the primary analysis, which was predominantly based on observational data.

Conversely, a sub-analysis restricted to RCTs demonstrated a significant 43% relative risk reduction of ATA recurrence associated with GA, indicating that GA may confer improved procedural efficacy in carefully selected patient populations.

These findings emphasize the necessity of individualized anaesthetic management. While sedation remains a viable and resource-efficient option for many patients, GA should be considered preferentially in complex cases where its procedural advantages may translate into enhanced clinical outcomes. Therefore, the choice of anaesthesia should be guided by patient-specific characteristics and procedural demands to optimize therapeutic efficacy.

Patient variability and methodological differences across studies also likely contributed to heterogeneity in outcomes. In Calvert *et al*. (1),^9^ patients with persistent AF were preferentially assigned to undergo ablation under GA (15.7% in the sedation group vs. 56.2% in the GA group), while Calvert *et al*. (2)^36^ restricted GA to high-risk patients, such as those with a body mass index ≥ 40, obstructive sleep apnoea, or other airway concerns. These methodological discrepancies, along with the smaller and more heterogeneous sample size in Calvert *et al*. (2),^36^ likely contribute to some of the variation observed.

Although no significant differences were observed in procedural, fluoroscopy, or ablation times, the potential cost implications of anaesthesia choice should not be overlooked. Two studies included in our analysis reported substantially lower costs associated with sedation compared to GA, consistent with expectations given the additional resources required for GA.^26,32^ However, these studies were conducted in England and China, and the generalizability of their findings may be limited by differences in healthcare systems and cost structures. Future cost-effectiveness studies across diverse healthcare settings are essential to better understand and address this balance, ensuring that the choice of anaesthesia aligns with both clinical outcomes and economic considerations.

Pulsed field ablation represents a promising advancement in AF management due to its unique non-thermal, tissue-selective properties, potentially enabling shorter procedures. However, the associated PFA-related pain raises uncertainty regarding the optimal sedation strategy. Recent studies have described the feasibility and tolerability of deep sedation protocols using spontaneous respiration, including those involving newer biphasic catheters and intravenous ketamine.^39,40^ Conference abstracts comparing GA with monitored anaesthesia care during PFA have found no significant differences in procedural times between groups; however, GA was associated with higher operator satisfaction, reduced operative pain, and minimized chest movements.^41^ Similarly, a recent prospective study showed no significant difference in procedural endpoints but reported substantially lower intra- and post-procedural pain associated with GA.^38^ These findings underscore the importance of balancing resource utilization and patient outcomes as PFA adoption continues to expand globally.^4,42^ Further studies are necessary to explore the impact of various sedation strategies on procedural efficiency, safety, and long-term outcomes, including ATA recurrence, among patients undergoing PFA.

Previous meta-analyses have suggested comparable safety and effectiveness between GA and sedation.^6,7^ However, those reviews included a smaller number of patients and relied predominantly on qualitative comparisons. In contrast, our meta-analysis incorporated over 7000 additional patients and multiple newly published studies, nearly quintupling the total sample size.^8,31,36^ We also conducted several sensitivity and meta-regression analyses, strengthening the reliability of our findings. Furthermore, we compared GA against all sedation strategies collectively, offering a broader and more contemporary perspective on anaesthesia choice during AF ablation.

Our findings provide comprehensive and contemporary evidence supporting the safety and efficacy of both sedation and GA for AF ablation. While sedation offers practical and economic advantages, GA remains an important option in selected patients. Future studies are warranted to refine patient selection criteria and evaluate outcomes in the evolving landscape of ablation technologies.

### Limitations

This study has several limitations. First, the predominance of observational studies in our overall analysis may inherently limit the strength and reliability of the pooled results. While our primary analysis shows a non-significant difference in recurrence rates between sedation and GA, we acknowledge that unmeasured confounding, common in observational data, could still influence this overall finding. However, to address potential biases, we conducted analysis restricted to RCTs, which revealed a statistically significant higher risk of ATA recurrence with sedation compared to GA. Second, some included studies were conducted many years ago, potentially affecting the relevance of our conclusions due to significant technological advancements in CA for AF. To mitigate this concern, we performed a meta-regression analysis based on publication year, which revealed no significant association. Third, the high heterogeneity observed in procedural, ablation, and fluoroscopy times likely reflects variations in protocols, operator experience, and endpoint definitions across studies. Therefore, these findings should be interpreted with caution. Fourth, the prevalence of persistent AF varied significantly across studies, which could have influenced the results of ATA recurrence. Fifth, differences in ablation strategies, such as the use of additional lesion sets beyond PVI, were not consistently reported and could not be accounted for in the analysis. We could not directly compare conscious vs. deep sedation due to the restricted number of studies reporting this comparison; therefore, we performed a subgroup analysis, indicating no subgroup interaction according to sedation strategy. Lastly, potential selection bias cannot be ruled out, as patients with more complex disease were more likely to receive GA. Although subgroup analyses with randomized and adjusted data helped mitigate this concern, residual confounding related to baseline patient differences may still influence the observed outcomes.

## Conclusion

In patients undergoing CA for AF, there was no significant difference in ATA recurrence between GA and sedation, regardless of sedation depth. However, given the potential for confounding by indication in observational studies, these findings should be interpreted with caution.

## Supplementary Material

euaf156_Supplementary_Data

## Data Availability

All data analysed in this meta-analysis are derived from previously published studies, which are cited throughout the manuscript. No new data were generated for this study.
