# Supplementary material for: Sedation vs. general anaesthesia in patients with atrial fibrillation undergoing catheter ablation: a systematic review and meta-analysis
Source: Europace. 2025 Sep 18;27(9):euaf156. doi: 10.1093/europace/euaf156 (PMC12448922; doi:10.1093/europace/euaf156)
Supplement: euaf156_Supplementary_Data [file euaf156_supplementary_data.docx]

**SUPPLEMENTARY MATERIAL**

**Sedation versus general anesthesia in patients with atrial fibrillation undergoing catheter ablation: a systematic review and meta-analysis**

**Table of contents**

[Supplemental Methods 1. PRISMA 2020 main checklist 3](#_Toc205472102)

[Supplemental Methods 2. PRISMA 2020 abstract checklist 7](#_Toc205472103)

[Supplemental Methods 3. Details of the Search Strategy 8](#_Toc205472104)

[Supplemental Methods 4. Agents used in sedation and general anesthesia 9](#_Toc205472105)

[Supplemental Methods 5. Definition and methods used by studies reporting recurrence of atrial tachyarrhythm. 11](#_Toc205472106)

[Supplemental Methods 6. Definition of procedural time, overall complications and anesthesia-related complications. 13](#_Toc205472107)

[Supplemental Figure 1. Subgroup analysis of recurrence of atrial tachyarrhythmia 17](#_Toc205472108)

[Supplemental Figure 2. Leave-one-out sensitivity analysis of recurrence of atrial tachyarrhythmia 22](#_Toc205472109)

[Supplemental Figure 3. Sensitivity analysis of recurrence outcome 23](#_Toc205472110)

[Supplemental Figure 4. Univariate meta-regression analyses for ATA recurrence 24](#_Toc205472111)

[Supplemental Figure 5. Subgroup Analyses of Complication Rates 29](#_Toc205472112)

[Supplemental Figure 6. Secondary endpoints. 31](#_Toc205472113)

[Supplemental Figure 7. Risk of bias assessment of recurrence of atrial tachyarrhythmia 33](#_Toc205472114)

[Supplemental Figure 8. Funnel plot and Egger’s regression test for the primary endpoint 35](#_Toc205472115)

# Supplemental Methods 1. PRISMA 2020 main checklist

| Topic | No. | Item | Location where item is reported |
| --- | --- | --- | --- |
| TITLE |  |  |  |
| Title | 1 | Identify the report as a systematic review. | Pg. 1 at MS |
| ABSTRACT |  |  |  |
| Abstract | 2 | See the PRISMA 2020 for Abstracts checklist | Pg. 2-3 |
| INTRODUCTION |  |  |  |
| Rationale | 3 | Describe the rationale for the review in the context of existing knowledge. | Pg. 5 at MS |
| Objectives | 4 | Provide an explicit statement of the objective(s) or question(s) the review addresses. | Pg. 5 at MS |
| METHODS |  |  |  |
| Eligibility criteria | 5 | Specify the inclusion and exclusion criteria for the review and how studies were grouped for the syntheses. | Pg 6 at MS |
| Information sources | 6 | Specify all databases, registers, websites, organizations, reference lists and other sources searched or consulted to identify studies. Specify the date when each source was last searched or consulted. | Pg 6 at MS |
| Search strategy | 7 | Present the full search strategies for all databases, registers, and websites, including any filters and limits used. | Pg 8 at sup |
| Selection process | 8 | Specify the methods used to decide whether a study met the inclusion criteria of the review, including how many reviewers screened each record and each report retrieved, whether they worked independently, and if applicable, details of automation tools used in the process. | Pg. 6 at MS |
| Data collection process | 9 | Specify the methods used to collect data from reports, including how many reviewers collected data from each report, whether they worked independently, any processes for obtaining or confirming data from study investigators, and if applicable, details of automation tools used in the process. | NA |
| Data items | 10a | List and define all outcomes for which data were sought. Specify whether all results that were compatible with each outcome domain in each study were sought (e.g. for all measures, time points, analyses), and if not, the methods used to decide which results to collect. | Pg. 7 at MS |
|  | 10b | List and define all other variables for which data were sought (e.g. participant and intervention characteristics, funding sources). Describe any assumptions made about any missing or unclear information. | NA |
| Study risk of bias assessment | 11 | Specify the methods used to assess risk of bias in the included studies, including details of the tool(s) used, how many reviewers assessed each study and whether they worked independently, and if applicable, details of automation tools used in the process. | Pg. 8 at MS |
| Effect measures | 12 | Specify for each outcome the effect measure(s) (e.g. risk ratio, mean difference) used in the synthesis or presentation of results. | Pg 8 at MS |
| Synthesis methods | 13a | Describe the processes used to decide which studies were eligible for each synthesis (e.g. tabulating the study intervention characteristics and comparing against the planned groups for each synthesis (item 5)). | NA |
|  | 13b | Describe any methods required to prepare the data for presentation or synthesis, such as handling of missing summary statistics, or data conversions. | Pg. 8 at MS |
|  | 13c | Describe any methods used to tabulate or visually display results of individual studies and syntheses. | Pg. 8 at MS |
|  | 13d | Describe any methods used to synthesize results and provide a rationale for the choice(s). If meta-analysis was performed, describe the model(s), method(s) to identify the presence and extent of statistical heterogeneity, and software package(s) used. | Pg. 8 at MS |
|  | 13e | Describe any methods used to explore possible causes of heterogeneity among study results (e.g. subgroup analysis, meta-regression). | Pg. 8 at MS |
|  | 13f | Describe any sensitivity analyses conducted to assess robustness of the synthesized results. | Pg. 8 at MS |
| Reporting bias assessment | 14 | Describe any methods used to assess risk of bias due to missing results in a synthesis (arising from reporting biases). | NA |
| Certainty assessment | 15 | Describe any methods used to assess certainty (or confidence) in the body of evidence for an outcome. | NA |
| RESULTS |  |  |  |
| Study selection | 16a | Describe the results of the search and selection process, from the number of records identified in the search to the number of studies included in the review, ideally using a flow diagram. | Figure 1 |
|  | 16b | Cite studies that might appear to meet the inclusion criteria, but which were excluded, and explain why they were excluded. | NA |
| Study characteristics | 17 | Cite each included study and present its characteristics. | NA |
| Risk of bias in studies | 18 | Present assessments of risk of bias for each included study. | NA |
| Results of individual studies | 19 | For all outcomes, present, for each study: (a) summary statistics for each group (where appropriate) and (b) an effect estimates and its precision (e.g. confidence/credible interval), ideally using structured tables or plots. | Fig. 1 at MS |
| Results of syntheses | 20a | For each synthesis, briefly summarise the characteristics and risk of bias among contributing studies. | NA |
|  | 20b | Present results of all statistical syntheses conducted. If meta-analysis was done, present for each the summary estimate and its precision (e.g. confidence/credible interval) and measures of statistical heterogeneity. If comparing groups, describe the direction of the effect. | NA |
|  | 20c | Present results of all investigations of possible causes of heterogeneity among study results. | NA |
|  | 20d | Present results of all sensitivity analyses conducted to assess the robustness of the synthesized results. | NA |
| Reporting biases | 21 | Present assessments of risk of bias due to missing results (arising from reporting biases) for each synthesis assessed. | NA |
| Certainty of evidence | 22 | Present assessments of certainty (or confidence) in the body of evidence for each outcome assessed. | NA |
| DISCUSSION |  |  |  |
| Discussion | 23a | Provide a general interpretation of the results in the context of other evidence. | Pg. 11-13 at MS |
|  | 23b | Discuss any limitations of the evidence included in the review. | Pg. 11-13 at MS |
|  | 23c | Discuss any limitations of the review processes used. | Pg. 11-13 at MS |
|  | 23d | Discuss implications of the results for practice, policy, and future research. | Pg. 11-13 at MS |
| OTHER INFORMATION |  |  |  |
| Registration and protocol | 24a | Provide registration information for the review, including register name and registration number, or state that the review was not registered. | PROSPERO; CRD42024589329 |
|  | 24b | Indicate where the review protocol can be accessed, or state that a protocol was not prepared. | https://www.crd.york.ac.uk/prospero/display_record.php?RecordID=589329 |
|  | 24c | Describe and explain any amendments to information provided at registration or in the protocol. | We performed an absolute risk assessment analysis. |
| Support | 25 | Describe sources of financial or non-financial support for the review, and the role of the funders or sponsors in the review. | None |
| Competing interests | 26 | Declare any competing interests of review authors. | Pg. 1 at MS |
| Availability of data, code and other materials | 27 | Report which of the following are publicly available and where they can be found: template data collection forms; data extracted from included studies; data used for all analyses; analytic code; any other materials used in the review. | NA |

^Abbreviations: MS, manuscript; NA, non-available; sup, supplemental material.^

# Supplemental Methods 2. PRISMA 2020 abstract checklist

| **Topic** | **No.** | **Item** | **Reported?** |
| --- | --- | --- | --- |
| **TITLE** |  |  |  |
| **Title** | 1 | Identify the report as a systematic review. | Yes |
| **BACKGROUND** |  |  |  |
| **Objectives** | 2 | Provide an explicit statement of the main objective(s) or question(s) the review addresses. | Yes |
| **METHODS** |  |  |  |
| **Eligibility criteria** | 3 | Specify the inclusion and exclusion criteria for the review. | Yes |
| **Information sources** | 4 | Specify the information sources (e.g. databases, registers) used to identify studies and the date when each was last searched. | Yes |
| **Risk of bias** | 5 | Specify the methods used to assess risk of bias in the included studies. | No |
| **Synthesis of results** | 6 | Specify the methods used to present and synthesize results. | Yes |
| **RESULTS** |  |  |  |
| **Included studies** | 7 | Give the total number of included studies and participants and summarise relevant characteristics of studies. | Yes |
| **Synthesis of results** | 8 | Present results for main outcomes, preferably indicating the number of included studies and participants for each. If meta-analysis was done, report the summary estimate and confidence/credible interval. If comparing groups, indicate the direction of the effect (i.e. which group is favoured). | Yes |
| **DISCUSSION** |  |  |  |
| **Limitations of evidence** | 9 | Provide a brief summary of the limitations of the evidence included in the review (e.g. study risk of bias, inconsistency and imprecision). | Yes |
| **Interpretation** | 10 | Provide a general interpretation of the results and important implications. | No |
| **OTHER** |  |  |  |
| **Funding** | 11 | Specify the primary source of funding for the review. | No |
| **Registration** | 12 | Provide the register name and registration number. | No |

| **Search Strategy for Each Database** | |
| --- | --- |
| **PubMed/**  **MEDLINE** | ("Atrial Fibrillation"[MeSH] OR AF OR Afib OR "A fib") AND (ablation OR isolation OR PVI OR "pulmonary vein isolation" OR "cryoablation" OR "cryoballoon ablation") AND ("Anesthesia, General"[MeSH] OR "General Anesthesia" OR "General Anaesthesia" OR GA) AND ("Sedation" OR "Conscious Sedation"[MeSH] OR "Moderate Sedation" OR CS OR MS OR "deep sedation" OR "mild sedation" OR "local anaesthesia" OR "local anesthesia" OR “monitored anesthesia” OR “monitored anaesthesia”) |
| **Cochrane Library** | ("Atrial Fibrillation" OR AF OR Afib OR "A fib") AND (ablation OR isolation OR PVI OR "pulmonary vein isolation" OR cryoablation OR "cryoballoon ablation") AND ("General Anesthesia" OR "General Anaesthesia" OR GA) AND ("Sedation" OR "Conscious Sedation" OR "Moderate Sedation" OR CS OR MS OR "deep sedation" OR "mild sedation" OR "local anesthesia" OR "local anaesthesia" OR "monitored anesthesia" OR "monitored anaesthesia") |
| **Embase** | ('atrial fibrillation'/exp OR 'AF' OR 'Afib' OR 'A fib') AND ('ablation'/exp OR 'isolation' OR 'PVI' OR 'pulmonary vein isolation' OR 'cryoablation' OR 'cryoballoon ablation') AND ('general anesthesia'/exp OR 'General Anesthesia' OR 'General Anaesthesia' OR 'GA') AND ('sedation'/exp OR 'conscious sedation'/exp OR 'Moderate Sedation' OR 'CS' OR 'MS' OR 'deep sedation' OR 'mild sedation' OR 'local anesthesia'/exp OR 'local anaesthesia' OR 'monitored anesthesia' OR 'monitored anaesthesia') |
| **ClinicalTrials.gov** | Condition or disease: "Atrial Fibrillation" OR AF OR Afib OR “A fib” Other terms: (ablation OR isolation OR PVI OR "pulmonary vein isolation" OR "cryoablation" OR "cryoballoon ablation") AND ("Anesthesia, General" OR "General Anesthesia" OR "General Anaesthesia" OR GA) AND ("Sedation" OR "Conscious Sedation"OR "Moderate Sedation" OR CS OR MS OR "deep sedation" OR "mild sedation" OR "local anaesthesia" OR "local anesthesia" OR “monitored anesthesia” OR “monitored anaesthesia”) |

# Supplemental Methods 3. Details of the Search Strategy

# Supplemental Methods 4. Agents used in sedation and general anesthesia

| **Study** | **Sedation** | **Anesthesia** |
| --- | --- | --- |
| **Bun, 2014** | Boluses of midazolam (maximum  5 mg) and nalbuphin (maximum 10 mg) | Titrated target-controlled infusion of propofol and remifentanil |
| **Calvert, 2024** | 25–50 μg fentanyl with or without  1–2 mg midazolam | NA |
| **Calvert, 2024**  **(2)** | Bolus doses of 1–2 mg midazolam and 50 mcg fentanyl. Doses could be adjusted based on operator discretion | A propofol bolus (1–2 mg/kg) and a muscle relaxant were administered, followed by anesthesia with a sevoflurane oxygen-air mixture, guided by continuous depth-of-anesthesia monitoring |
| **Chikata, 2017** | 6 μg/kg/h dexmedetomidine for 10 min, followed by 0.2–0.7 μg/kg/h | 1–2 mg/kg propofol and  1–2 μg/kg fentanyl, followed by  3–6 mg/kg/h propofol |
| **Di Biase, 2011** | Fentanyl and midazolam | 2 mg/kg propofol,  fentanyl 1–2 μg/kg, and  0.6–1 mg/kg rocuronium |
| **Di Biase, 2009** | Fentanyl or midazolam | 1 mg/kg propofol followed by a maintenance dose of 4 mg kg1 h1 to achieve adequate relief from pain. |
| **Firme, 2012** | 0.5 mg/kg midazolam, 1 mg/kg propofol followed by 25–50 μg/kg/min,  0.01–0.05 mg/kg/min remifentanil | 0.5 mg/kg midazolam, 2 mg/kg propofol followed by 60–100 μg/kg/min,  0.06–0.1 mg/kg/min remifentanil |
| **Kanthasamy, 2023** | Midazolam and diamorphine | NA |
| **Kuno, 2023** | 1‒5 μg/kg/h dexmedetomidine  followed by 0.15‒0.3 μg/kg/h,  50‒100 mg fentanyl, followed by 1‒5 μg/kg/h | 1‒2 mg/kg propofol,  0.05‒0.2 μg/kg/min remifentanil  0.6‒0.9 mg/kg rocuronium,  and 4‒5% desflurane |
| **Lo, 2025** | NA | NA |
| **Mahmoodi, 2023** | Fentanyl and midazolam | Midazolam, propofol, remifentanil,  and a paralyzing agent |
| **Martin, 2017** | Fentanyl and midazolam | 2–3 mg/kg propofol and  0.02–0.3 μg/kg/min remifentanil |
| **Minciună, 2024** | Morphine and midazolam | 2 mg/kg propofol,  1–2 μg /kg fentanyl, and  0.6–1 mg/kg rocuronium |
| **Moravec, 2021** | 100 μg fentanyl and  0.03-0.2 mg/kg/h midazolam | 0.5 μg g/kg sufentanil,  1-2 mg/kg propofol,  0.6 mg/kg rocuronium, and sevoflurane |
| **Riis-Vestergaard, 2024** | NA | NA |
| **Rillig, 2024** | Fentanyl 25 μg  and Propofol (1% solution) followed by a continuous Propofol infusion. | Remifentanil, propofol and succinylcholine or rocuronium |
| **Sochorová, 2025** | Concious sedation arm:  Midazolam IV bolus, sufentanil (5–10 µg), propofol loading (0.8–1.0 mg/kg) + boluses (0.5 mg/kg as needed). If inadequate: extra midazolam/sufentanil.  Deep sedation arm:  Remimazolam loading dose followed by continuous infusion (0.5 mg/kg/h based on IBW); ketamine bolus (1 mg/kg IBW) before ablation | Propofol-based TIVA with a secured airway |
| **Stašková, 2017** | Supracaine (local),  1 mg midazolam,  100 μg fentanyl | 0.12–0.15 mg/kg etomidate or  0.15 mg/kg propofol,  1–2 mg midazolam,  5–10 μg sufentanil,  0.4–0.6 mg/kg atracurium,  and 0.8–1.5% sevoflurane |
| **Wang, 2021** | Fentanyl and midazolam | 2–3 mg/kg propofol,  0.02–0.3 μg/kg/min remifentanil,  and a paralyzing agent |
| **Wang, 2024** | Intravenous dexmedetomidine and fentanyl, and local anesthesia with lidocaine | 2 mg/kg propofol,  0.2–0.5 μg/kg sufentanil,  0.6 mg/kg rocuronium,  and 1–2% sevoflurane |
| **Wasserlauf, 2016** | Intravenous boluses of midazolam and fentanyl | Opioid followed by a neuromuscular  blocker |
| **Wasserlauf, 2020** | Intravenous boluses of midazolam and fentanyl | Opioid followed by a neuromuscular blocker |
| **Xu, 2017** | Lidocaine 2% is used for local anesthesia, while fentanyl 0.3 mg, midazolam 10 mg, and 0.9% saline are administered | For induction: midazolam 2 mg, Etomidate 0.2 mg/kg, Sufentanil 0.3 µg/kg, and Cisatracurium 0.15 mg/kg. For maintenance: Remifentanil infusion at 0.1–0.5 µg/kg/h and Propofol 4–7 mg/kg/h |
| **Yokowaka, 2022** | 25−100 mcg/kg/min propofol with 25−200 mcg fentanyl  or continuous low dose infusion 0.5−2 mg remifentanil | For induction: Fentanyl 25–200 µg and Rocuronium 0.6–1.2 mg/kg.  For maintenance: Propofol 50–200 µg/kg/min or inhaled volatile anesthetics at 0.5–2 minimum alveolar concentration |

Abbreviations: TIVA: total intravenous anesthesia.

# Supplemental Methods 5. Definition and methods used by studies reporting recurrence of atrial tachyarrhythm.

| **Author, year** | **Atrial tachyarrhythmia** | **Method and definition** | **Blanking period** |
| --- | --- | --- | --- |
| **Bun, 2014** | AF or AT | Symptom-guided visits at the outpatient clinic and two 7-day Holters (6 months and 12 months). Recurrence was defined as a documented AF or left AT episode lasting ≥ 30 seconds. | 3-month blanking period |
| **Calvert, 2024**  **(1)** | AF | 12-lead ECGs at 4- and 12-months post ablation and any unscheduled symptom triggered telephone encounters with our Heart Rhythm Specialist Nurse, symptom-guided visits, and in some cases, patients utilize their own wearable devices, and traces are emailed and uploaded to our patient records for review. Recurrence was defined as a documented arrhythmia recurrence up to 12 months post ablation. |  |
| **Calvert, 2024**  **(2)** | AF | Determined from review of electronic patient records. | NA |
| **Chikata, 2017** | AF or AT | 12-lead electrocardiogram and 24-h Holter-electrocardiogram. Recurrence was defined as any episode of AF/AT lasting ≥ 30 seconds. | 3-month blanking period |
| **Di Biase, 2011** | AF or AT | The follow-up was performed at 3, 6, 9, and 12 months after the procedure, with a cardiology evaluation, a 12-lead electrocardiogram, and 7-day Holter monitoring. All patients received an event recorder for the first 5 months to record any symptomatic events. In addition, random recordings were performed 2 to 3 times per week to monitor for any asymptomatic episodes of AF. Recurrence was defined as any episode of AF/AT lasting for at least 30 seconds. | 2-month blanking period |
| **Firme, 2012** | AF | The monitoring consisted of a 12-lead electrocardiogram, pulse oximetry, capnography, and non-invasive blood pressure. A sensor was placed in the dorsal region to locate the position of the left atrium, which served as a guide for the electroanatomic mapping system to construct the left atrial geometry. | 3-month blanking period |
| **Mahmoodi, 2023** | - | All patients were reviewed as a routine at 3 months with a  Holter monitor and clinic visit, followed by clinic visits and Holter  monitors at 6, and 12 months. |  |
| **Martin, 2017** | Atrial arrhythmia | Clinical assessments, 12 lead ECGs and where appropriate 24 h Holter monitors, were obtained at baseline and at 3, 6, 12, and 18 months after the ablation. |  |
| **Minciună, 2024** | AF or AT | Follow-up evaluations were conducted at 3- and 6-months post-procedure and included a cardiology assessment, a 12-lead electrocardiogram, and 24 h Holter monitoring. Recurrence was defined as any episode of AF/AT lasting more than 30 s. | - |
| **Moravec, 2021** | Arrhythmia | Every 3 months after RFA (3, 6, 9 and 12 months after RFA, a 7-days ambulatory ECG monitoring was done in all patients. | 3-months blanking period |
| **Riis-Vestergaard, 2024** | AF | Defined as a composite endpoint of either admission, redeemed prescription of AAD, electric cardioversions or re-ablation during follow-up. |  |
| **Stašková, 2017** | AF or AT | Patients underwent 7-day Holter monitoring and clinical reviews at 3, 6, and 12 months. |  |
| **Wang, 2024** | Atrial tachyarrhythmia | Clinical evaluation and 24-h Holter recordings were performed at 1, 3, 6, and 12 months after the procedure. The 12-lead surface ECG was performed at any time if the patients reported arrhythmic symptoms. Recurrence was defined as atrial tachyarrhythmia lasting over 30 s during the follow-up. |  |
| **Wang, 2021** | Atrial tachyarrhythmia | Follow-up was performed at 1, 3, 6, 9, and 12 months using a 12-lead electrocardiogram at each visit. A 24-hour Holter monitoring was performed at 3, 6, 9, and 12 months. For non-paroxysmal AF, 7-day Holter monitoring was performed at 6 months. Recurrence was defined as any atrial tachyarrhythmia lasting ≥ 30 s. |  |
| **Wasserlauf, 2016** | AF, AT, or atrial flutter | Rhythm follow-up included, at a minimum, a 3-week AF monitor performed off AAD at 3 months post-procedure, and 24 to 48-hour Holter monitors thereafter at 6-month intervals up to two years, or downloads from implanted devices when available. Additional monitoring was performed in response to patient symptoms. Surface ECGs were obtained at each clinic visit. Recurrence was defined as AF, atrial flutter, and atrial tachycardia of any duration on surface ECG or >30 sec on rhythm monitoring without requirement for antiarrhythmic medication. |  |
| **Xu, 2017** | AF, AT, or atrial flutter | Patients were followed up by phone at 1, 3-, 6-, 9-, and 12-months post-procedure to assess for recurrence of atrial arrhythmias. The timing of any recurrences was recorded, and 12-lead ECGs and 24-hour Holter monitoring were performed. Patients reporting symptoms such as palpitations or chest discomfort were advised to seek immediate consultation for an ECG. |  |
| **Yokowaka, 2022** | Atrial tachyarrhythmia | The rhythm status was monitored using an auto‐triggered event monitor, serial electrocardiograms, and extended Holter monitors routinely at 6−12 months after RFA and whenever symptoms suggestive of an arrhythmia were reported. Recurrence was defined as any symptomatic or asymptomatic atrial tachyarrhythmia lasting >30 s. |  |

**Abbreviations:** AF: atrial fibrillation; AT: atrial tachycardia; LA: left atrium; NA: not available; RFA: radiofrequency ablation.

# Supplemental Methods 6. Definition of procedural time, overall complications and anesthesia-related complications.

| **Author, year** | **Procedural time** | **Laboratory occupancy time** | **Overall complications** | **Anesthesia-related complications** |
| --- | --- | --- | --- | --- |
| **Bun, 2014** | NA | NA | Defined as death, atrioesophageal fistula, pulmonary vein stenosis requiring intervention, pericardial tamponade, systemic embolic events, phrenic nerve paralysis, femoral vessel damage requiring surgery, blood transfusion, or prolongation of hospitalization. | There were no GA-related specific complications. Not specified. |
| **Calvert, 2024**  **(1)** | Skin‐to‐skin time refers to the time from first needle puncture to sheath removal. | Total cath lab time refers to the time from the patient entering to exiting the lab. | A single complication (major femoral vascular injury requiring surgical intervention) occurred in the 50 W/GA group, despite ultrasound‐guided access. No map shifts were recorded in either cohort. | NA |
| **Calvert, 2024**  **(2)** | NA | Total procedure time incorporated the time from patient entering to leaving the catheter lab. | No complications or adverse events were observed. | NA |
| **Chikata, 2017** | Procedure time was defined as the interval from draping of the patient until removal of catheter sheaths from the patients. | Total laboratory time was defined as the time from entrance to exit in the catheterization laboratory. | Defined as a pericardial effusion, stroke, phrenic nerve injury, atrioesophageal fistula, or any other major adverse events. | NA |
| **Di Biase, 2009** | NA | NA | Periprocedural complications. | NA |
| **Di Biase, 2011** | Procedure starts and finish times were defined as from draping of the patient to catheter removal from the body. | NA | Defined as pericardial effusion, stroke or transient ischemic attack, phrenic nerve injury, atrioesophageal fistula, or any other major complications. | No complication related to the anesthetic agents or to the  general anesthesia was described in this patient population. |
| **Firme, 2012** | NA | NA | Agitation or FA cardioversion or hemopericardium. | Cough leading to movement. |
| **Kanthasamy, 2023** | Skin to skin time. | NA | Procedure-related. | NA |
| **Kuno, 2022** | Entry-to-exit time. | NA | Periprocedural complications, such as stroke, cardiac tamponade, symptomatic severe PV stenosis, atrioesophageal fistula, or reintubation. | NA |
| **Lo, 2025** | Including the protocol-mandated 20-minute waiting period, pre-and post-procedure phrenic nerve stimulation, and pre-and post-procedure voltage mapping. | NA | NA | NA |
| **Mahmoodi, 2023** | NA | NA | Procedural or anesthetic complication rates. | There were no sedation-related complications in the GA group. In the CS group, one patient with morbid obesity and OSA developed type 2 respiratory failure post-procedure, likely due to peri-procedural phenergan for contrast allergy, requiring non-invasive ventilation. Another patient in the CS group became agitated and confused after anesthesia. |
| **Martin, 2017** | NA | Total laboratory time was not explicitly defined; inferred from discussion as time from entrance to exit, including anesthesia. | NA | Five procedures under sedation were hindered by airway problems, patients becoming agitated or with uncontrolled pain. |
| **Minciună, 2024** | Total procedural time. | NA | Intra- and post-procedural  complications. | There were no complications related to the general anesthesia or the anesthetic agents  used in this patient population. |
| **Moravec, 2021** | Time from when the patient laid on the operation table until he or she was transferred into a hospital bed fully conscious. | Time from when the patient laid on the operation until he or she was transferred into a hospital bed  fully conscious. | Cardiac tamponade, atrio-esophageal fistula, femoral artery pseudoaneurysm, transient ischemic attack, stroke or any other severe complication. | NA |
| **Rillig, 2024** | Total procedure time. | Defined was laboratory occupancy time. | Cardiac tamponade, stroke or transitory ischemic attack, air embolism, clinically apparent coronary spasm, phrenic nerve paralysis. | NA |
| **Riis-Vestergaard, 2024** | Procedure duration excludes anesthesia time. | NA | Included major adverse events such as stroke or admission for heart failure. | NA |
| **Sochorová, 2025** | Total procedure time (skin-to-skin, i.e., skin opening through skin closing) | NA | Was a composite of hypoxemia, hypotensive, or hypertensive events requiring intervention (such as increased FiO2, airway management, or drug administration) or resulting in procedure discontinuation. | Ketamine-related  Agitation/complication related to anesthesia. |
| **Stašková, 2017** | Total procedure time. | NA | Cardiac tamponade, fistula atrioventricular. | NA |
| **Wang, 2024** | NA | NA | NA | NA |
| **Wang, 2021** | Procedural time included the anesthesia induction time. | NA | NA | NA |
| **Wasserlauf, 2016** | Period from injection of lidocaine until removal of catheters. | Total time in laboratory (sum of procedure and non-procedure time). | They defined complications as significant adverse events occurring after the procedures, including bleeding at the groin puncture site, right phrenic nerve palsy, urinary tract infections and arteriovenous fistula at 30 days. | NA |
| **Wasserlauf, 2021** | NA | NA | NA | There were no cases of respiratory depression in the MS group including any need for pharmacologic reversal agents, conversion to general anesthesia, or bag valve mask ventilation. |
| **Xu, 2017** | NA | NA | NA | NA |
| **Yokowaka, 2022** | Procedure-start to procedure-end. | Mean duration of anesthesia care (Use anesthesia care duration as the best estimate for total lab time whenever possible). | Included cardiac tamponade, pericarditis with pericardial effusion, transient ischemic attack, cerebral embolic event, phrenic nerve palsy, groin hematoma, arteriovenous fistula, pseudoaneurysm, and aspiration pneumonia. | Aspiration pneumonia. |

**Abbreviations:** GA: general anesthesia; NA: not available; MS: moderate sedation

# Supplemental Figure 1. Subgroup analysis of recurrence of atrial tachyarrhythmia

**Figure 1A.** Recurrence of atrial tachyarrhythmia according to sedation type.

**
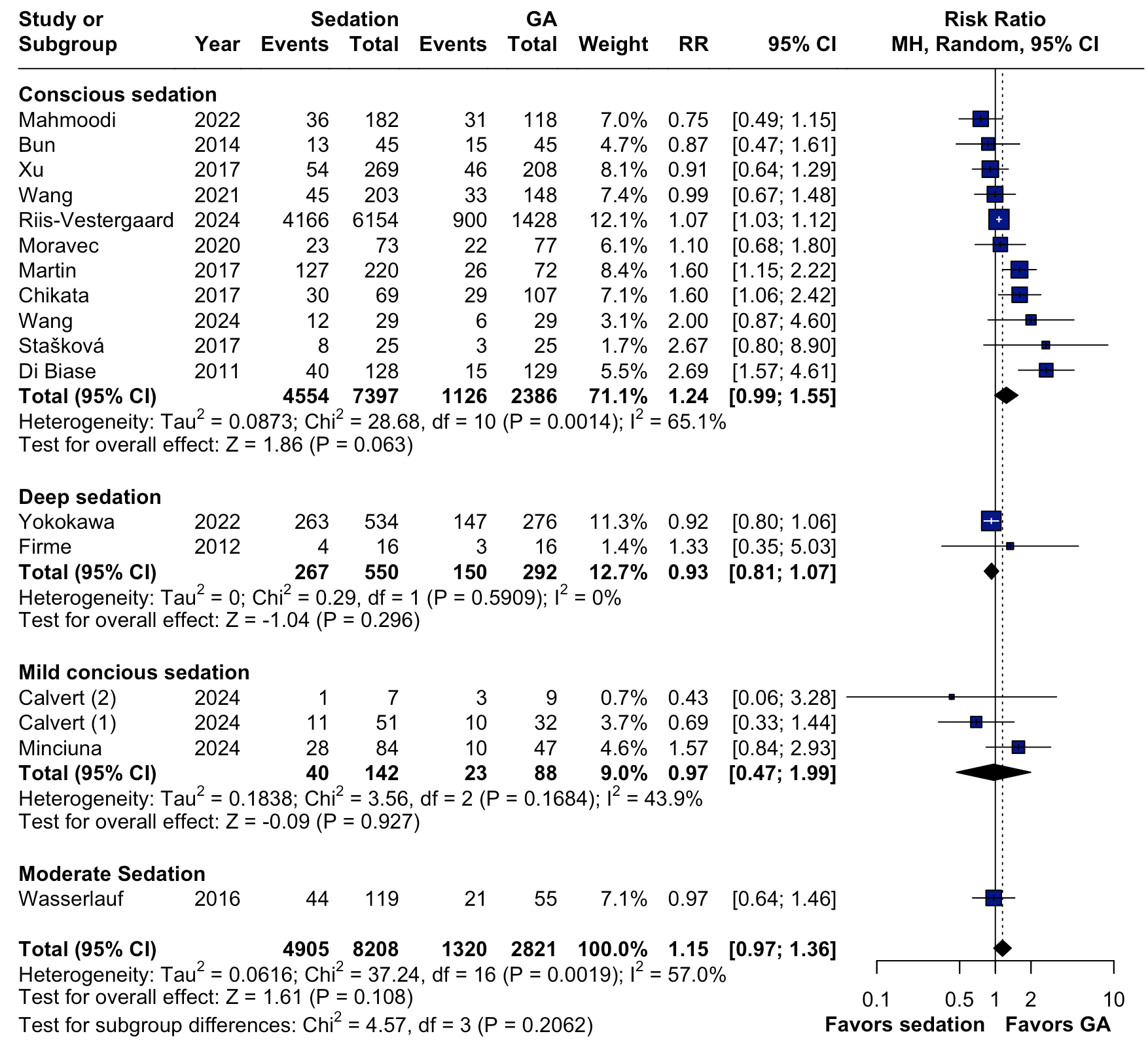
**

**Figure 1B.** Recurrence of atrial tachyarrhythmia according to study design.


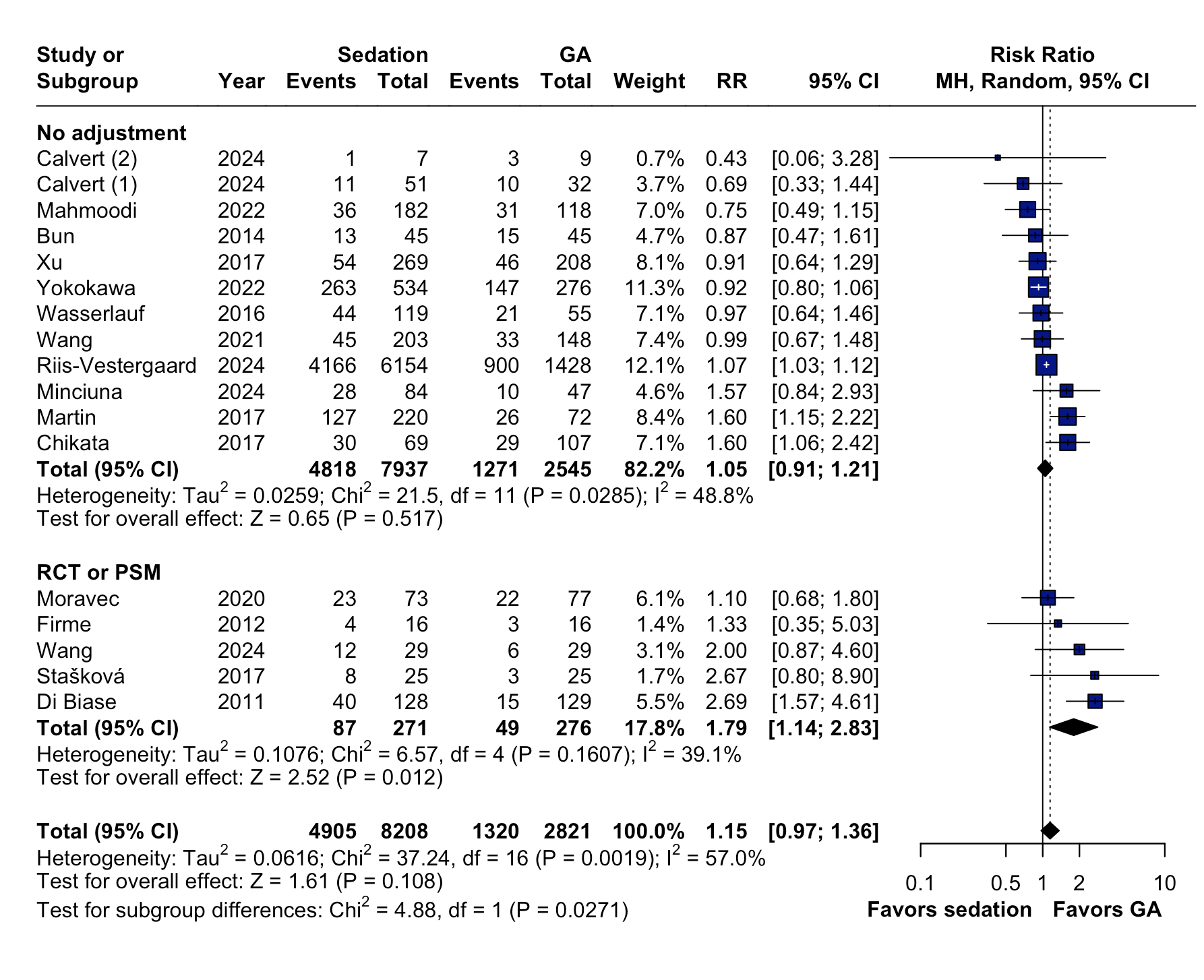
**Figure 1C.** Recurrence of atrial tachyarrhythmia according to energy source.


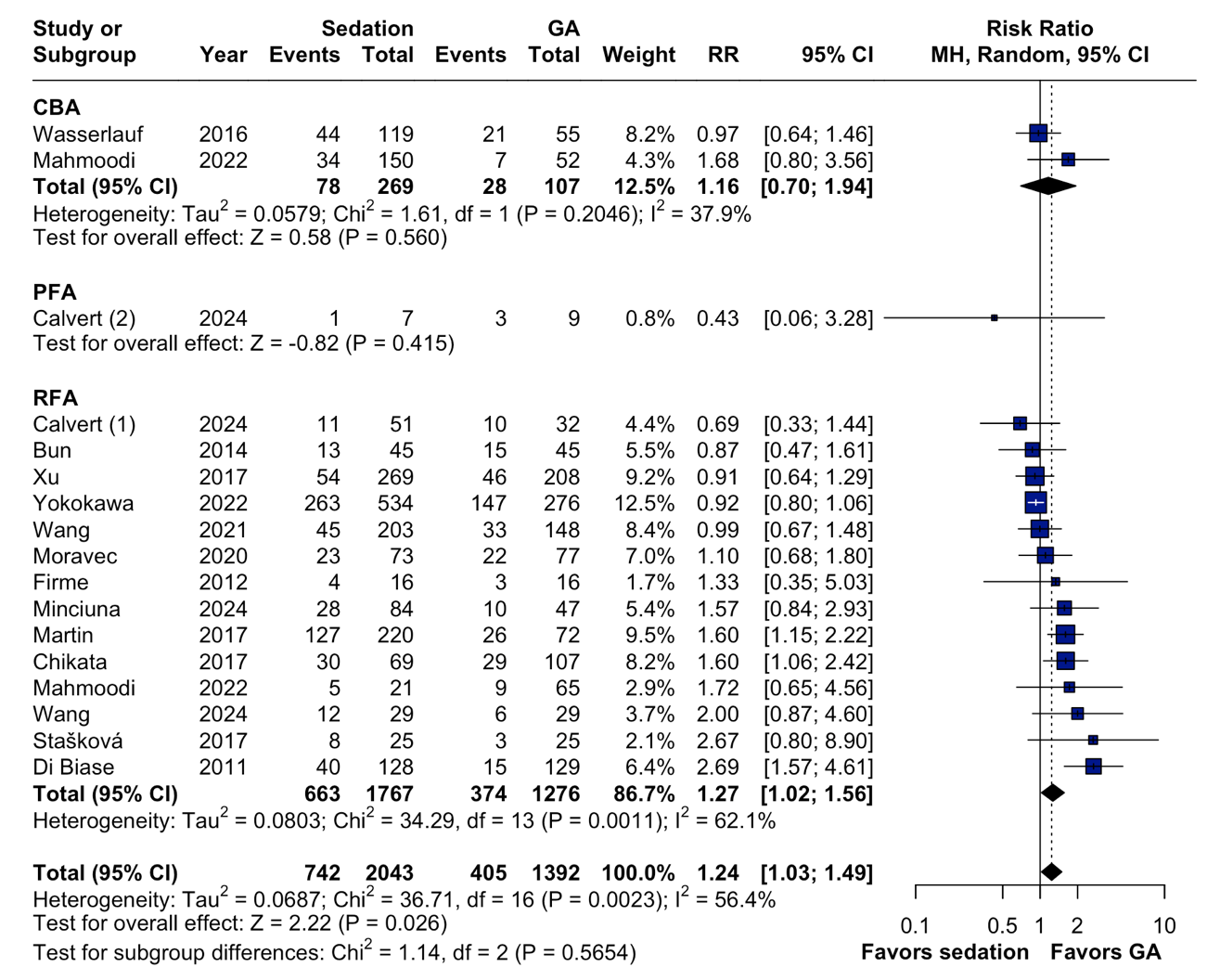


**Figure 1D.** Recurrence of atrial tachyarrhythmia according to study continent.


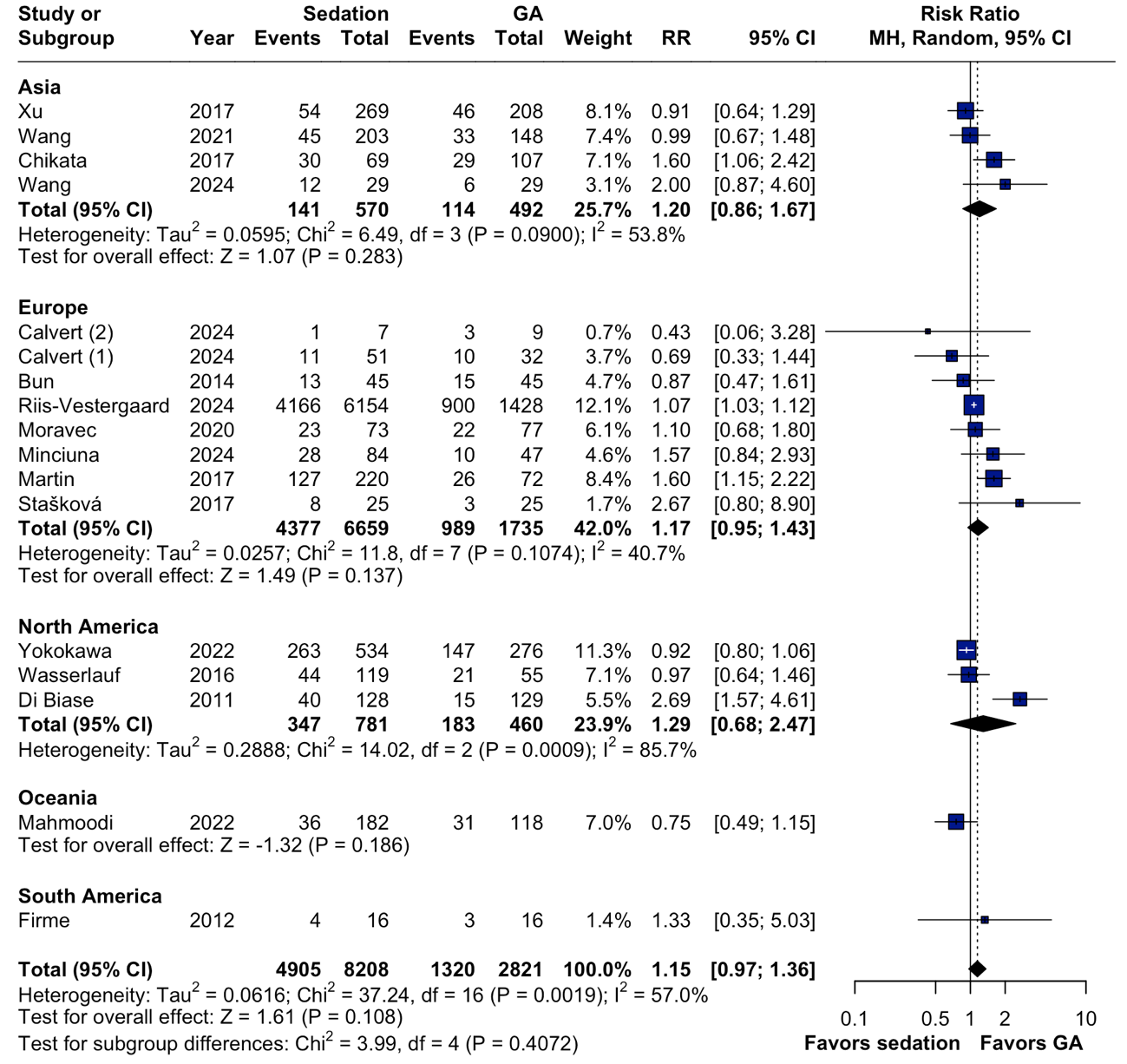


**Figure 1E.** Recurrence of atrial tachyarrhythmia according to ablation strategy.

**
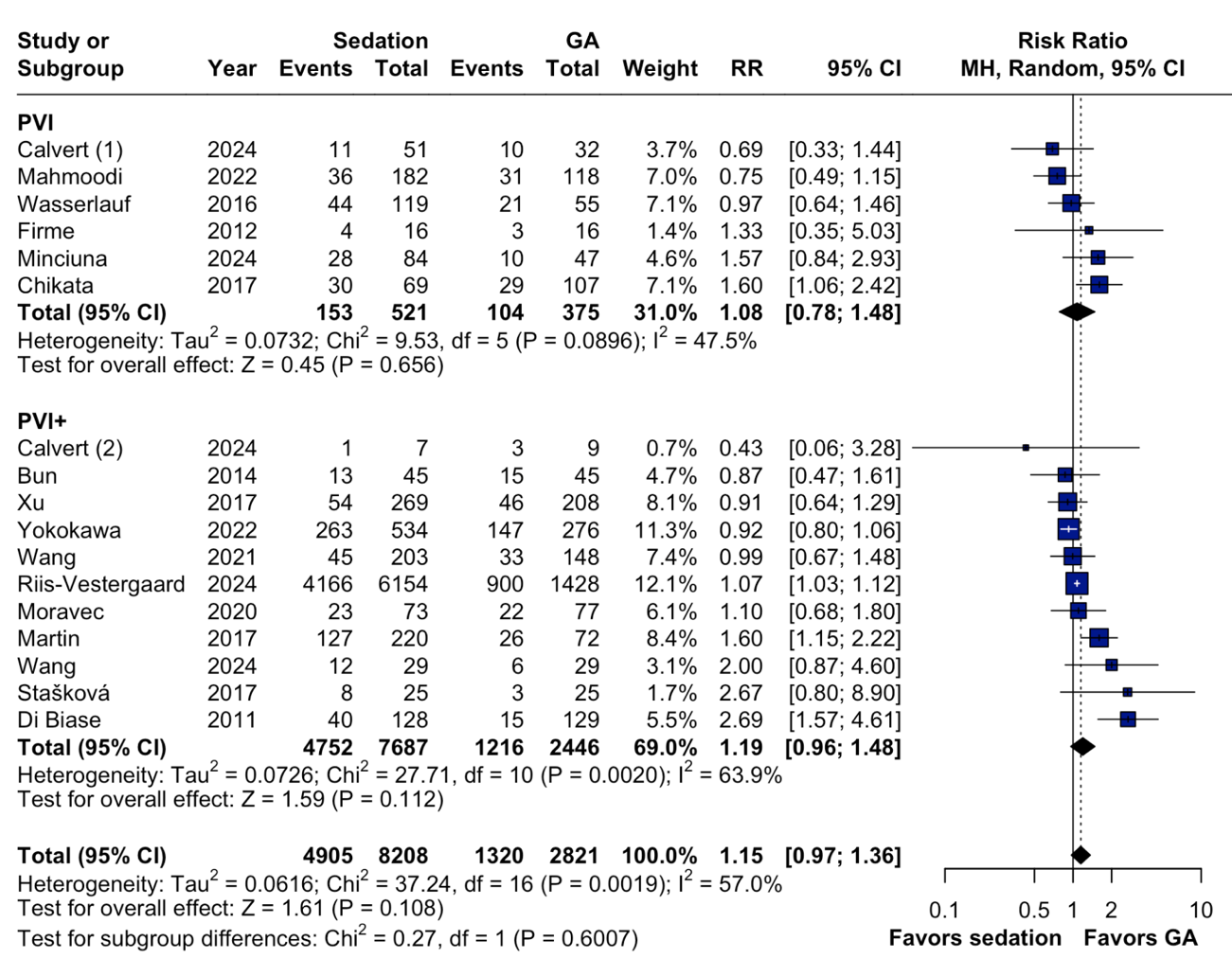
**

# Supplemental Figure 2. Leave-one-out sensitivity analysis of recurrence of atrial tachyarrhythmia


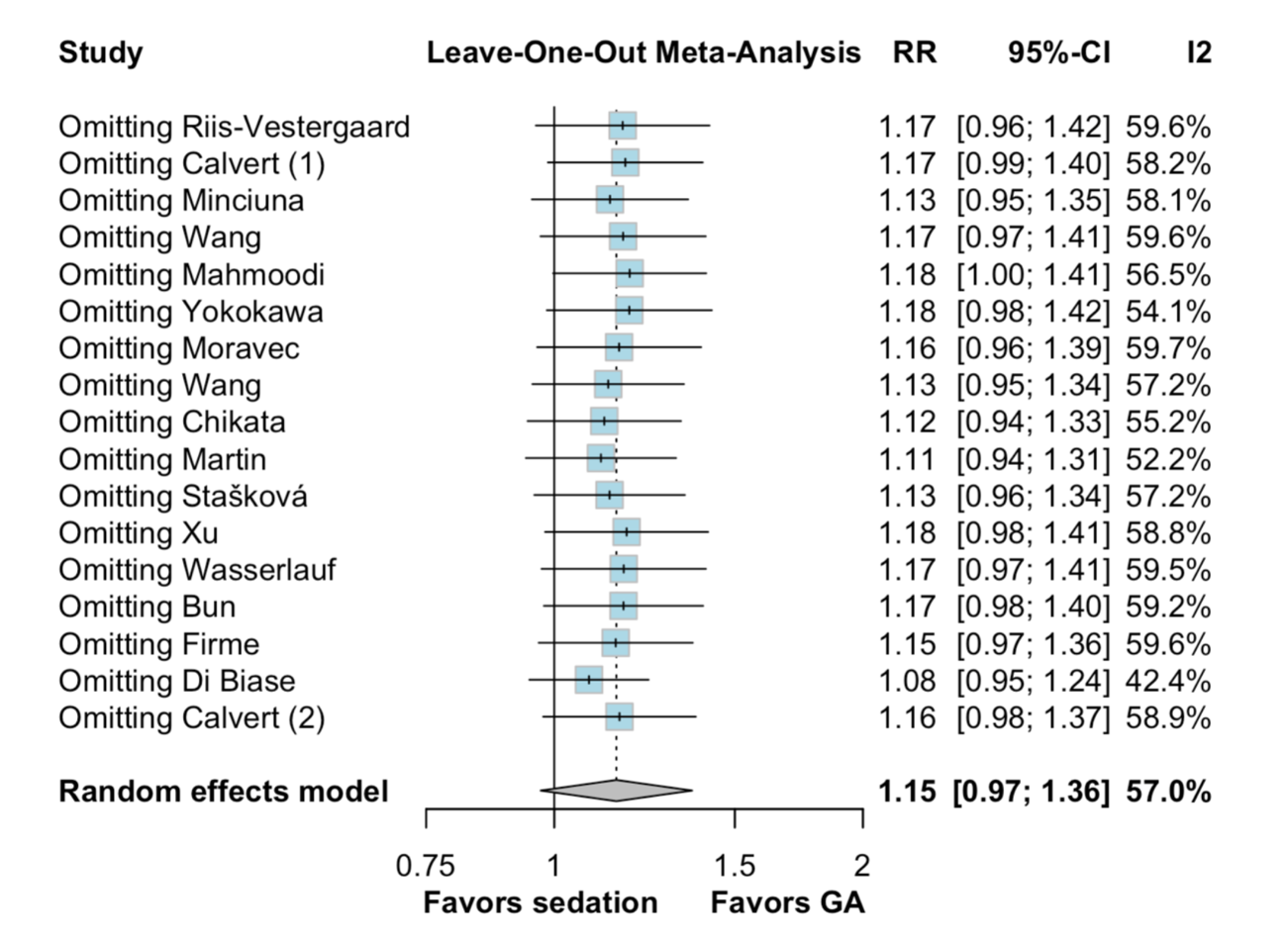


# Supplemental Figure 3. Sensitivity analysis of recurrence outcome

**Figure 3A.** Recurrence analysis restricted to RCTs


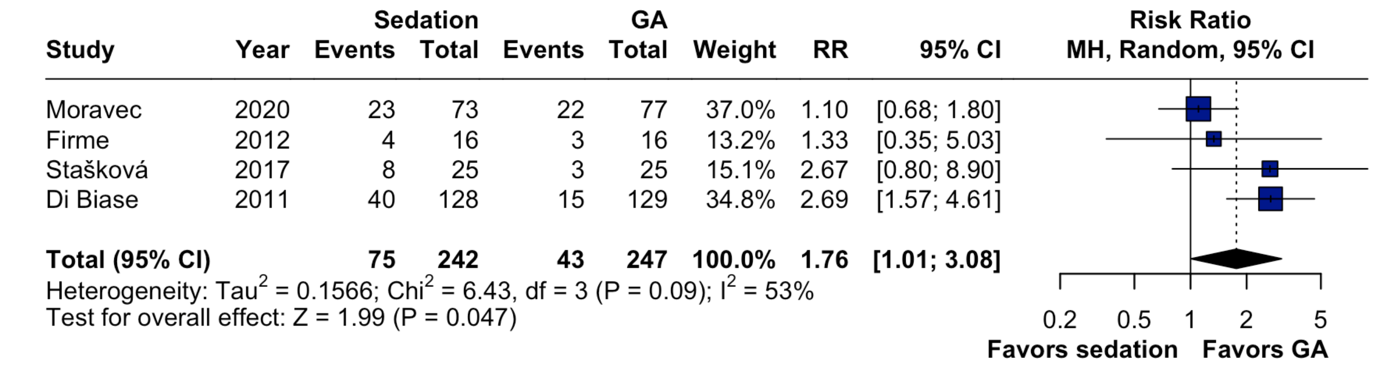


**Figure 3B.** Recurrence analysis with extended follow-up (17–60 months)


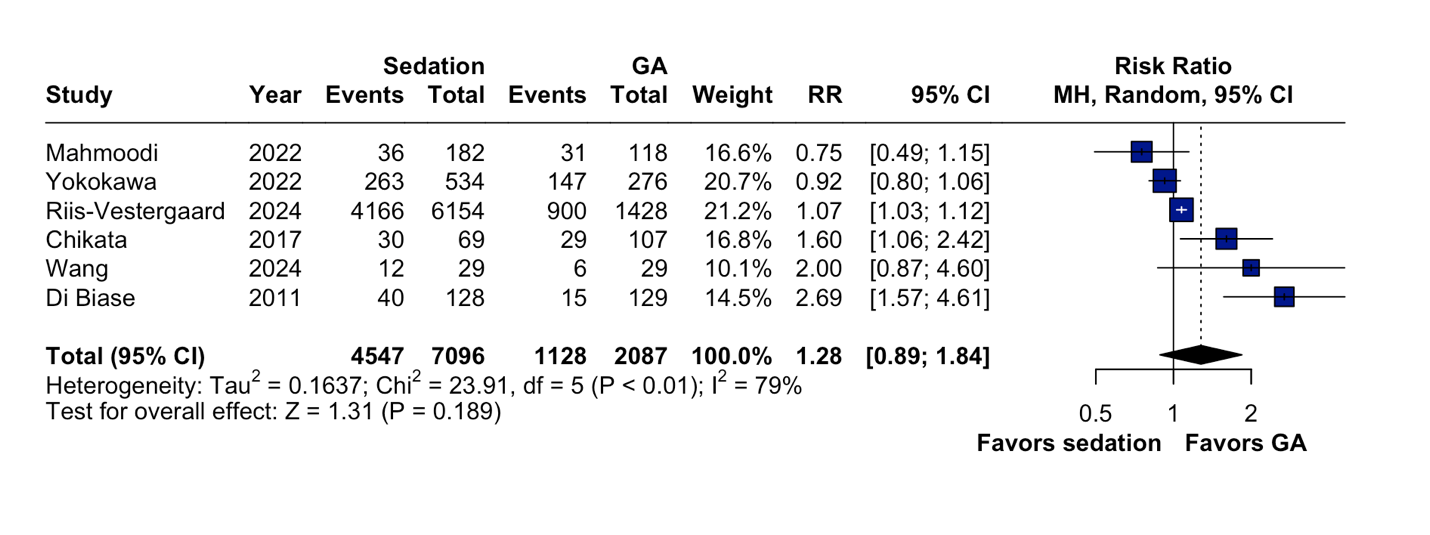


# Supplemental Figure 4. Univariate meta-regression analyses for ATA recurrence

**Figure 4A**. Meta-regression and bubble plot evaluating the association between follow-up duration and ATA recurrence in studies reporting this covariate.


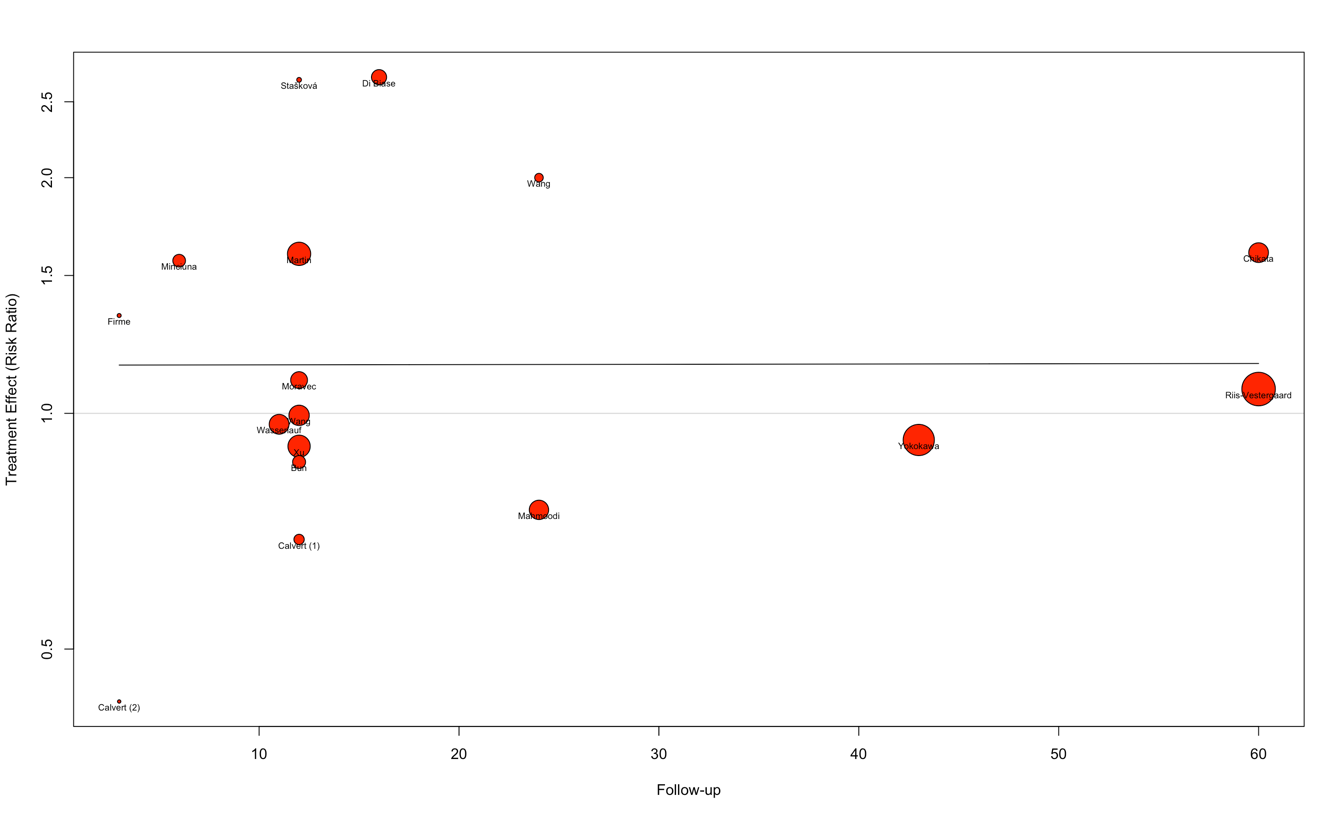


|  | Effect Estimate | p-value | R^2^ | Test for Residual Heterogeneity |
| --- | --- | --- | --- | --- |
| Intercept | 0.2231 | 0.1283 | 0.0% | 67.4% |
| Follow-up | [-0.0037] | 0.4836 |  |  |

**Figure 4B.** Meta-regression and bubble plot assessing the association between ATA recurrence and hypertension prevalence.


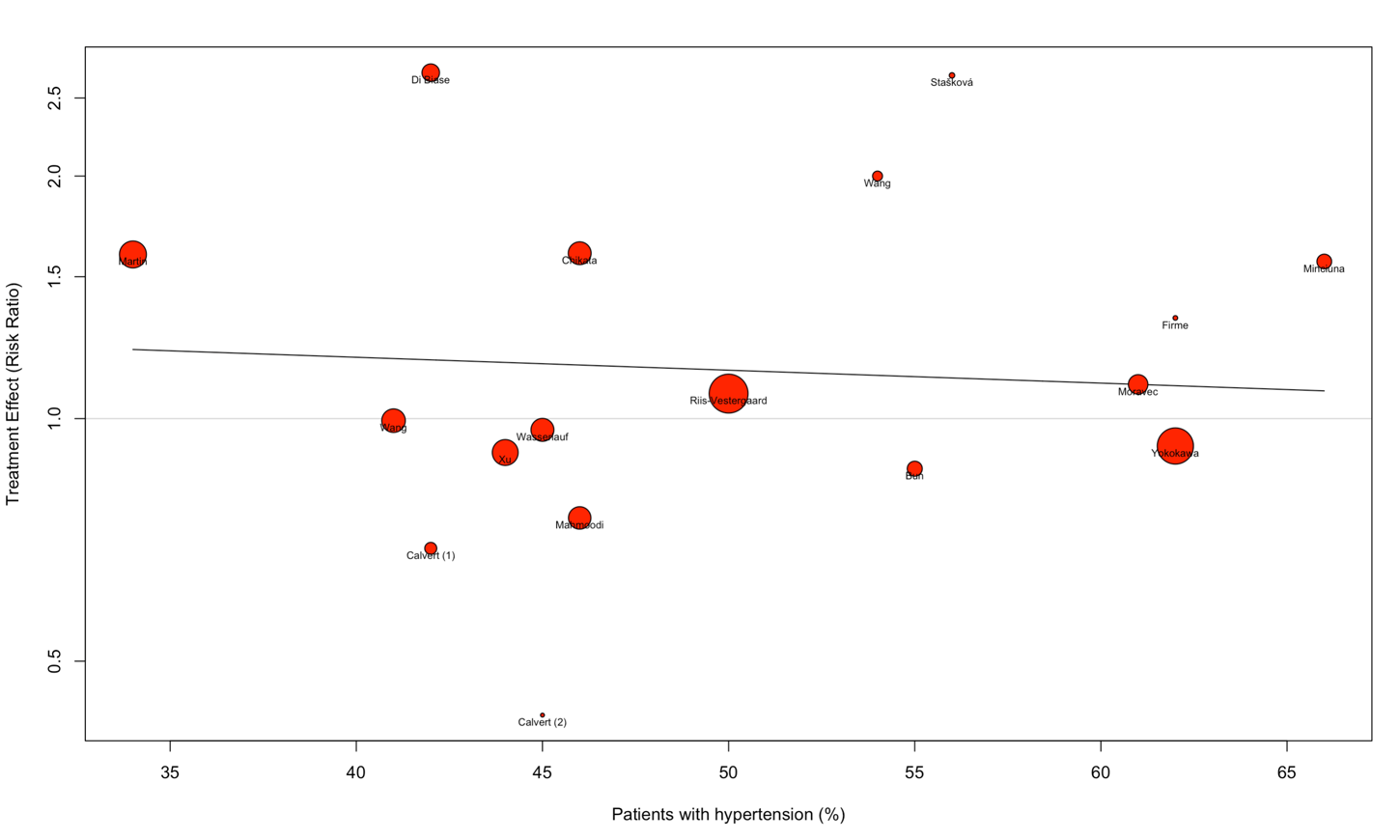


|  | Effect Estimate | p-value | R^2^ | Test for Residual Heterogeneity |
| --- | --- | --- | --- | --- |
| Intercept | 0.3230 | 0.5176 | 0.0% | 73.19% |
| Hypertension | [-0.037] | 0.7114 |  |  |

**Figure 4C.** Meta-regression and bubble plot assessing the association between ATA recurrence and participants’ mean age.

**
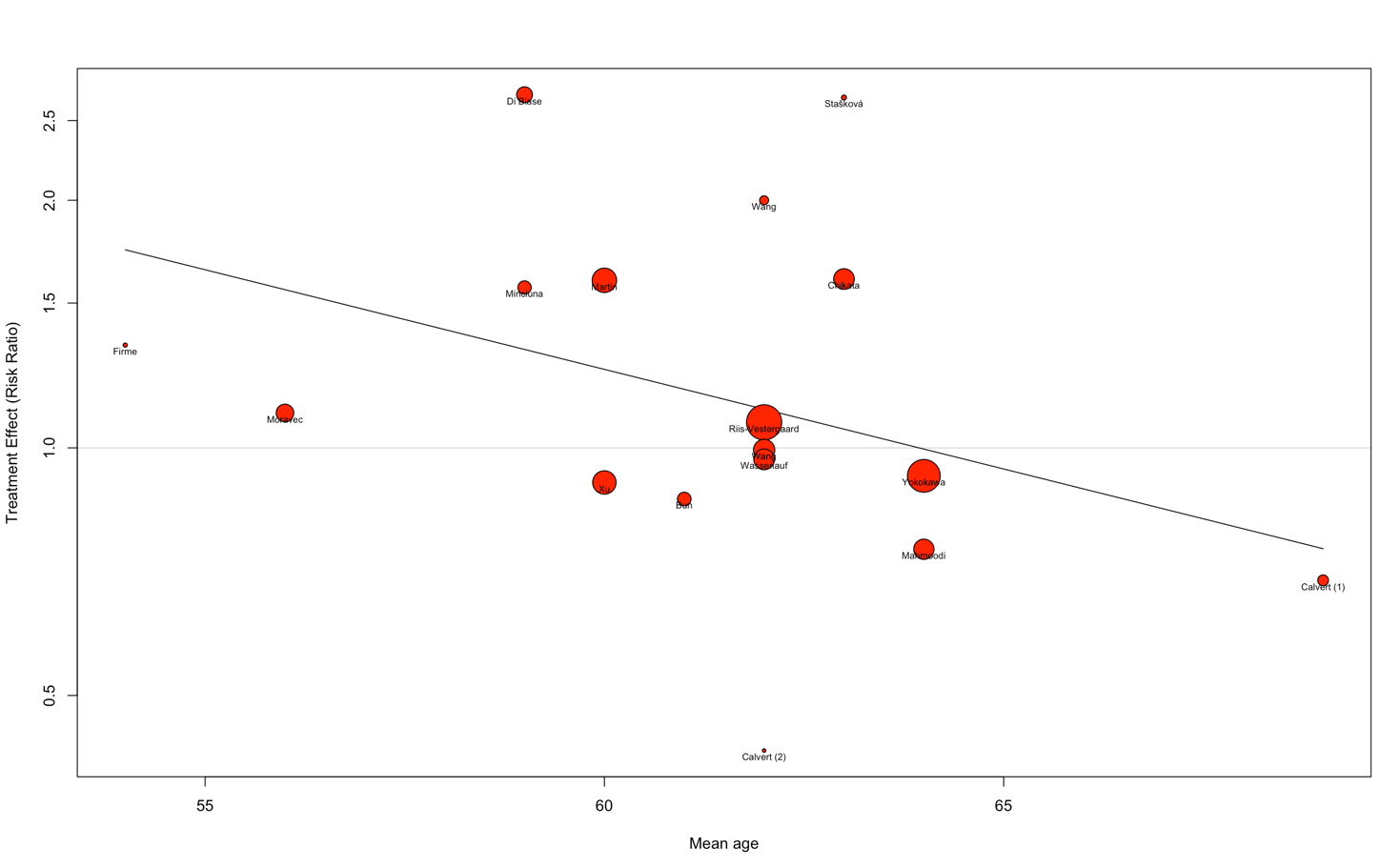
**

|  | Effect Estimate | p-value | R^2^ | Test for Residual Heterogeneity |
| --- | --- | --- | --- | --- |
| Intercept | 3.5666 | 0.0490 | 37.91% | 63.29% |
| Mean age | [-0.0558] | 0.0576 |  |  |

**Figure 4D.** Meta-regression and bubble plot assessing the association between ATA recurrence and the year of publication.

**
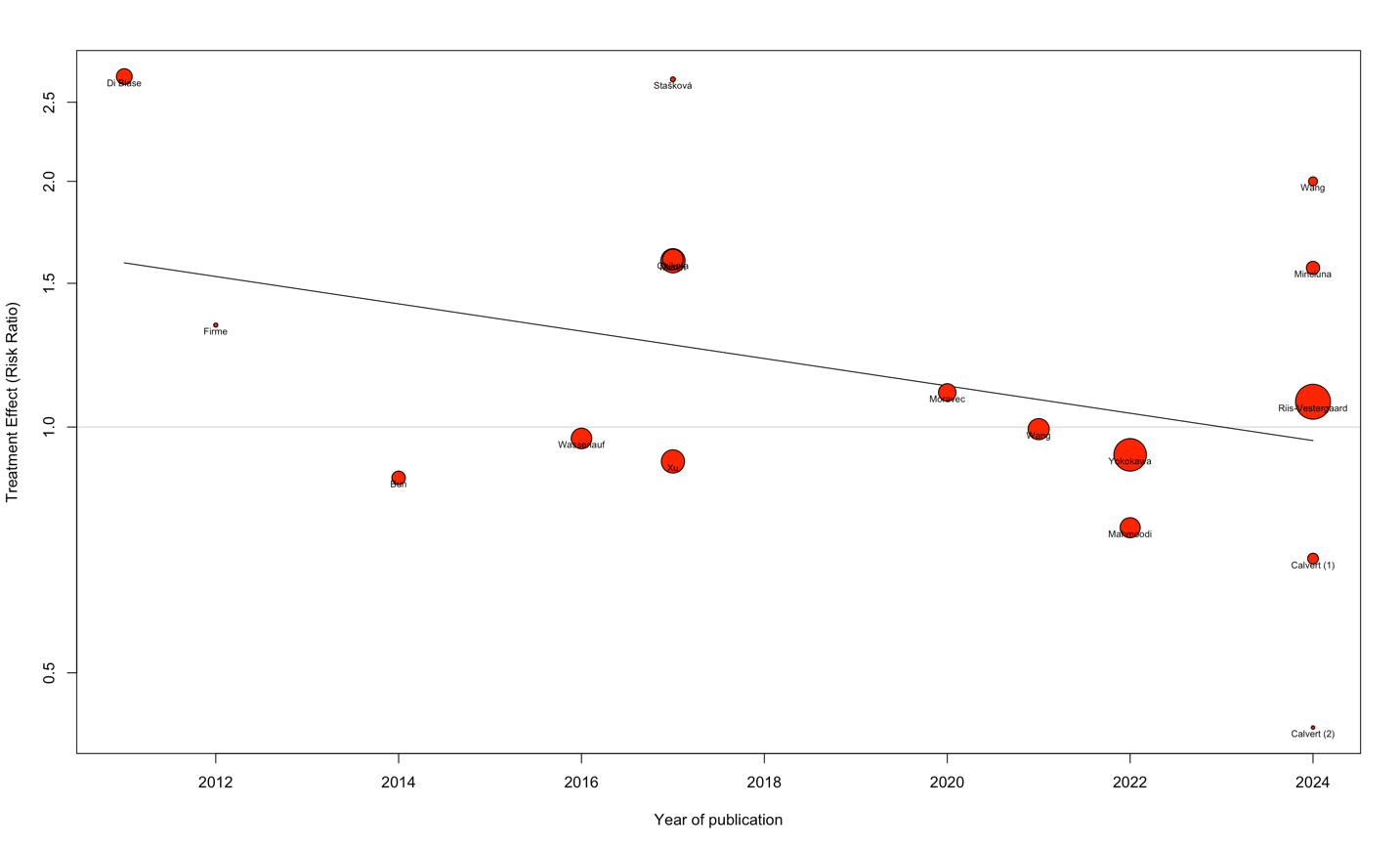
**

|  | Effect Estimate | p-value | R^2^ | Test for Residual Heterogeneity |
| --- | --- | --- | --- | --- |
| Intercept | 78.0068 | 0.0567 | 33.1% | 59% |
| Year of publication | [-0.0386] | 0.0571 |  |  |

**Figure 4E.** Meta-regression and bubble plot assessing the association between ATA recurrence and proportion of males.


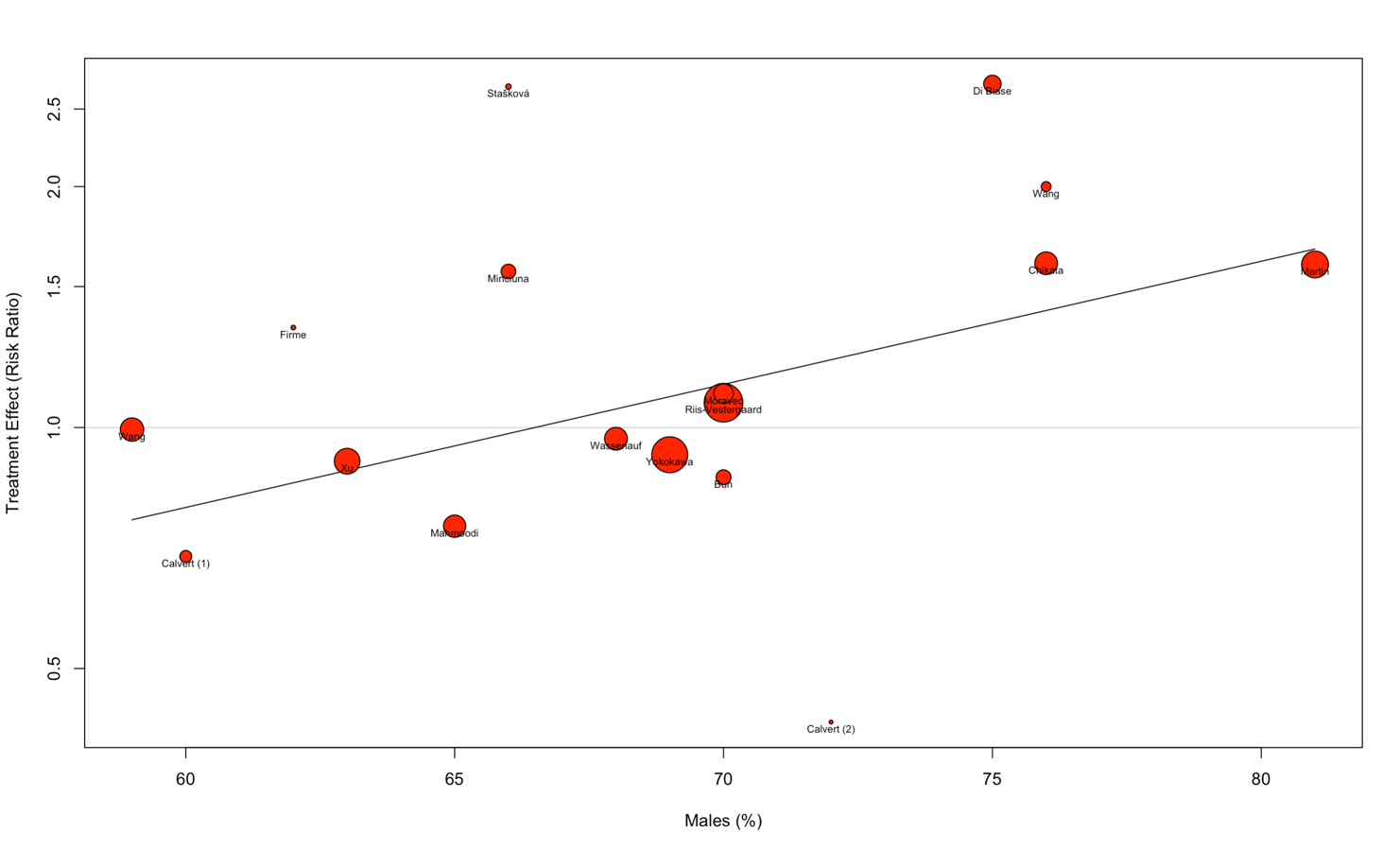


|  | Effect Estimate | p-value | R^2^ | Test for Residual Heterogeneity |
| --- | --- | --- | --- | --- |
| Intercept | [-2.3545] | 0.0014 | 80.4% | 36.72% |
| Proportion of males | 0.0354 | 0.0008 |  |  |

# Supplemental Figure 5. Subgroup Analyses of Complication Rates

**Figure 5A**. Anesthesia-related complications


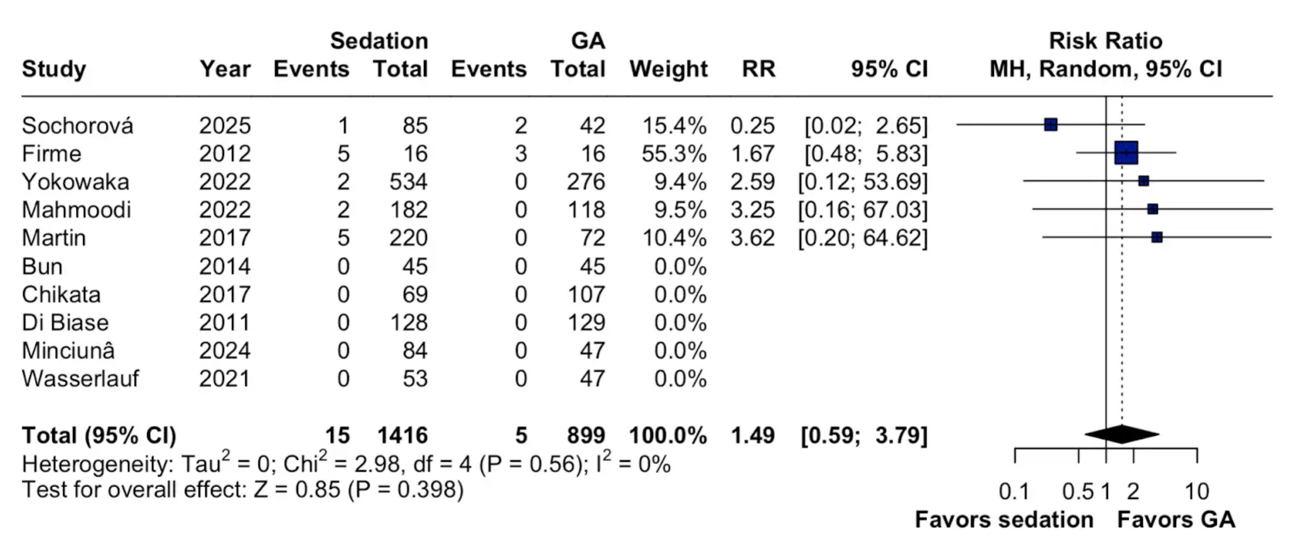


**Figure 5B**. Overall complications according to ablation strategy.

**
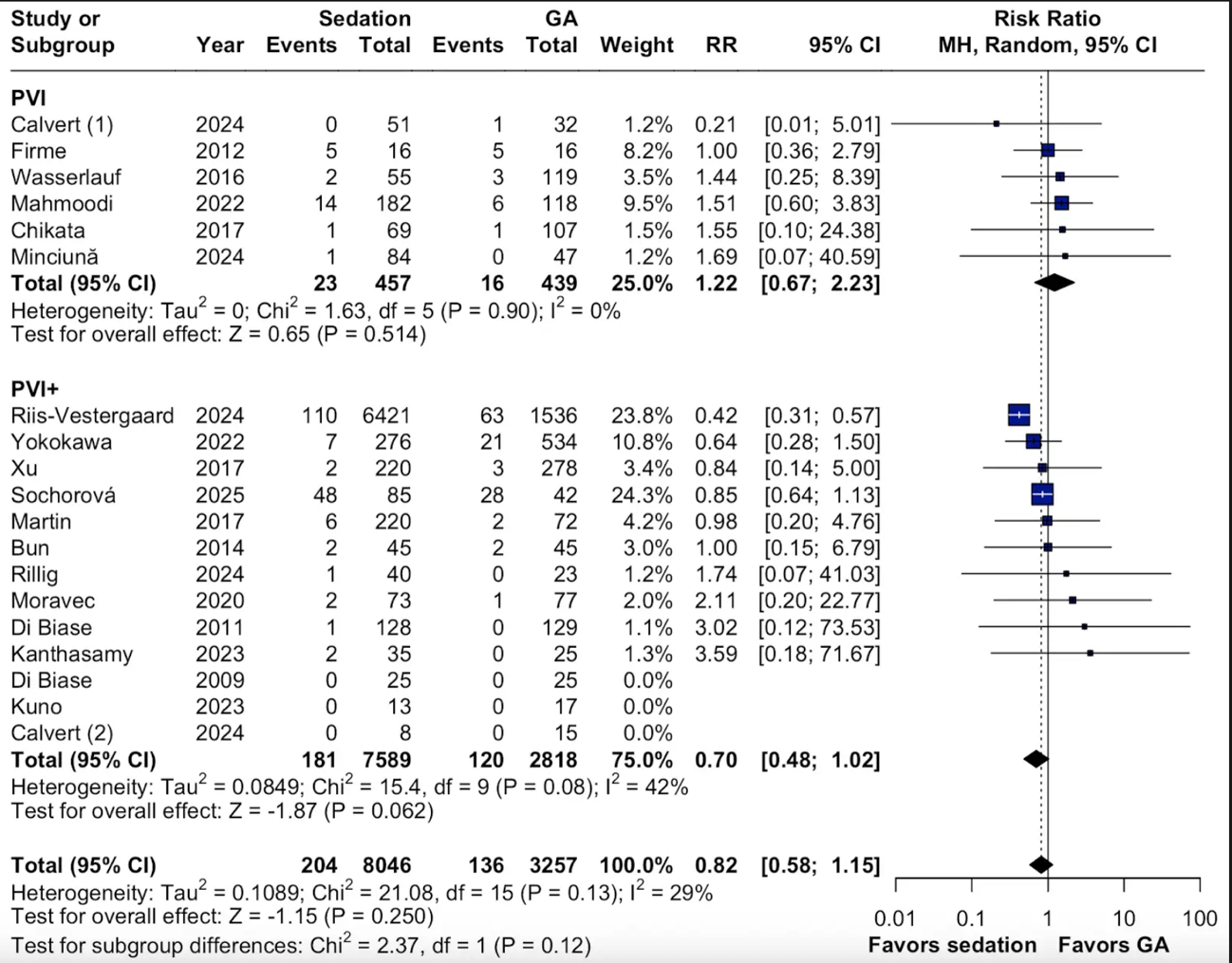
**

**Figure 5C.** Overall complications according to energy source.


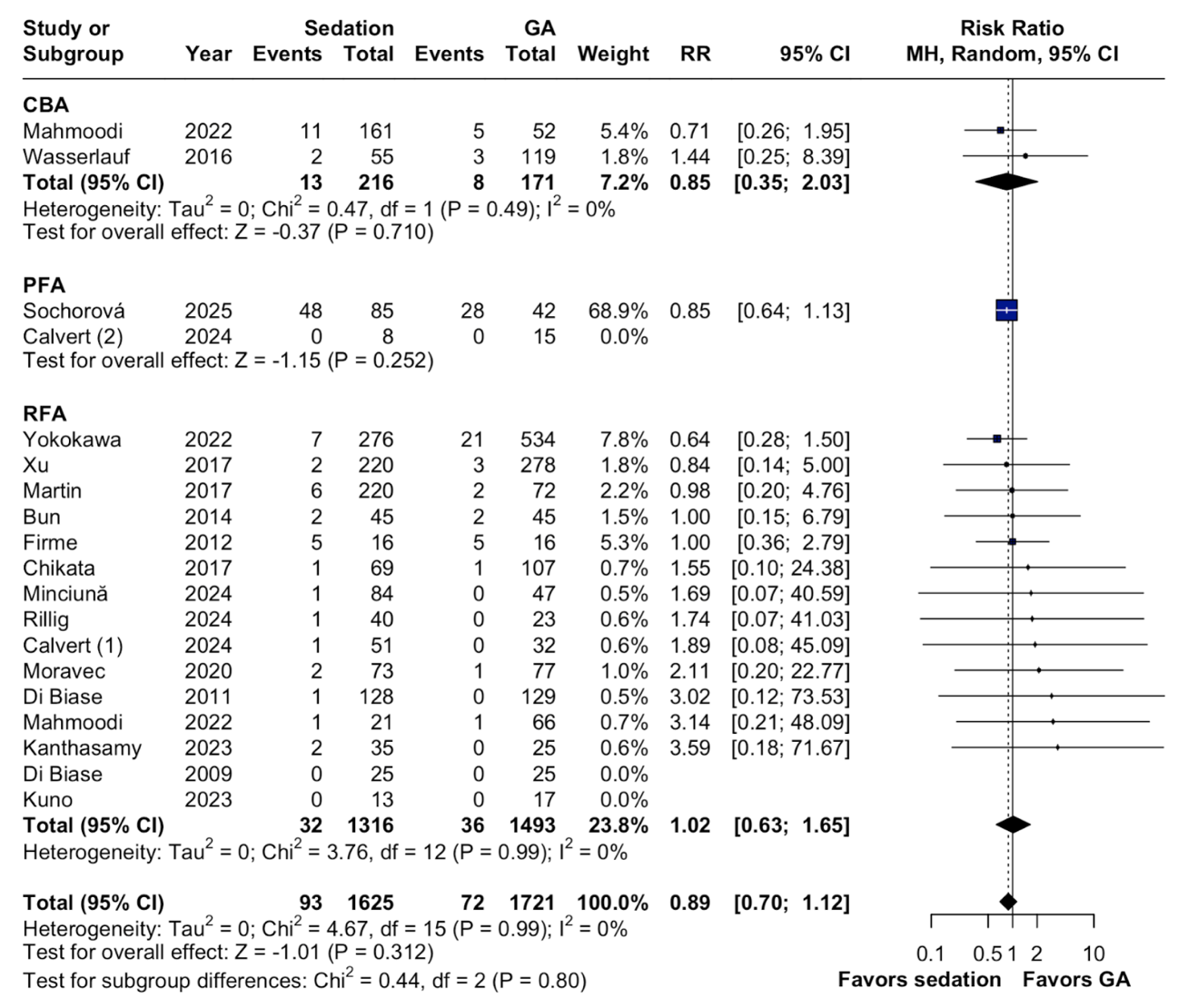


# Supplemental Figure 6. Secondary endpoints.

**Figure 6A.** Procedural duration

**
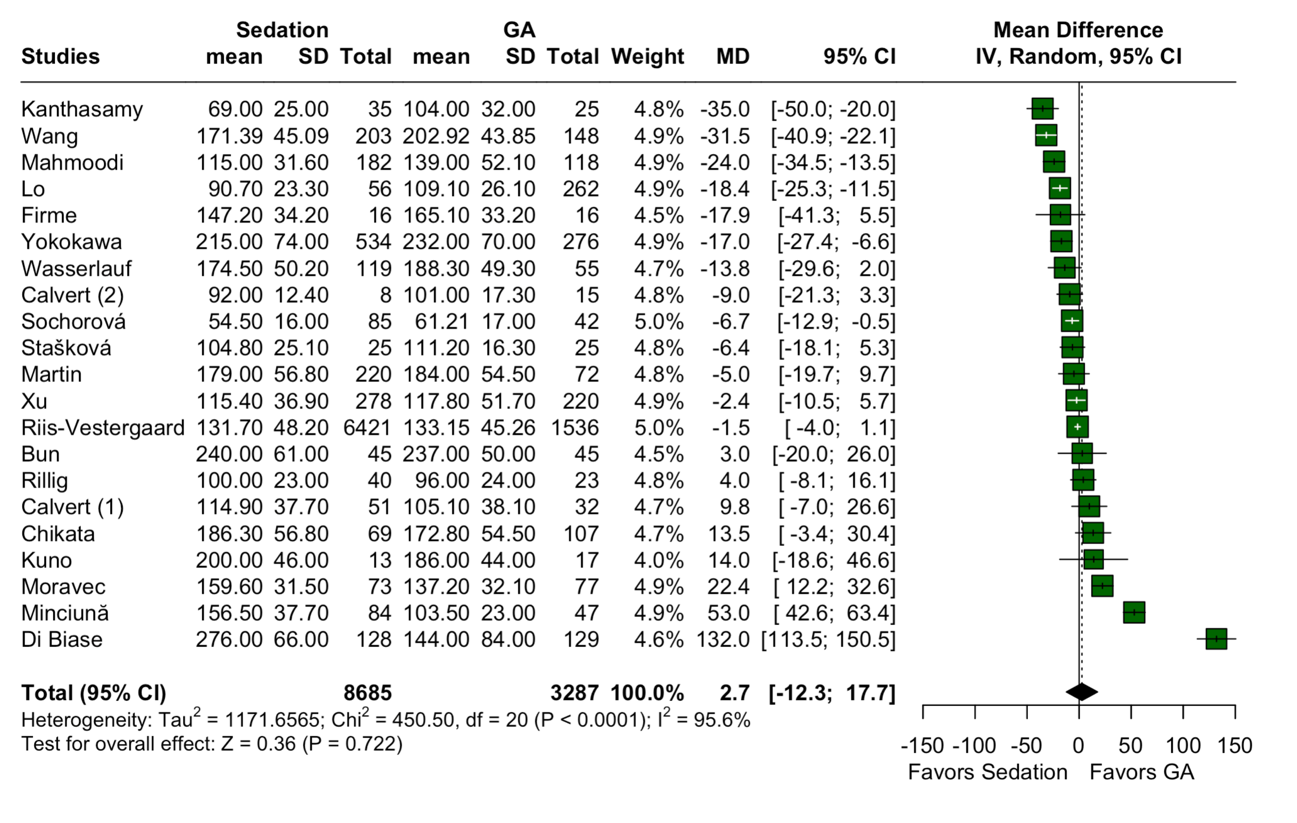
**

**Figure 6B.** Fluoroscopy time

**
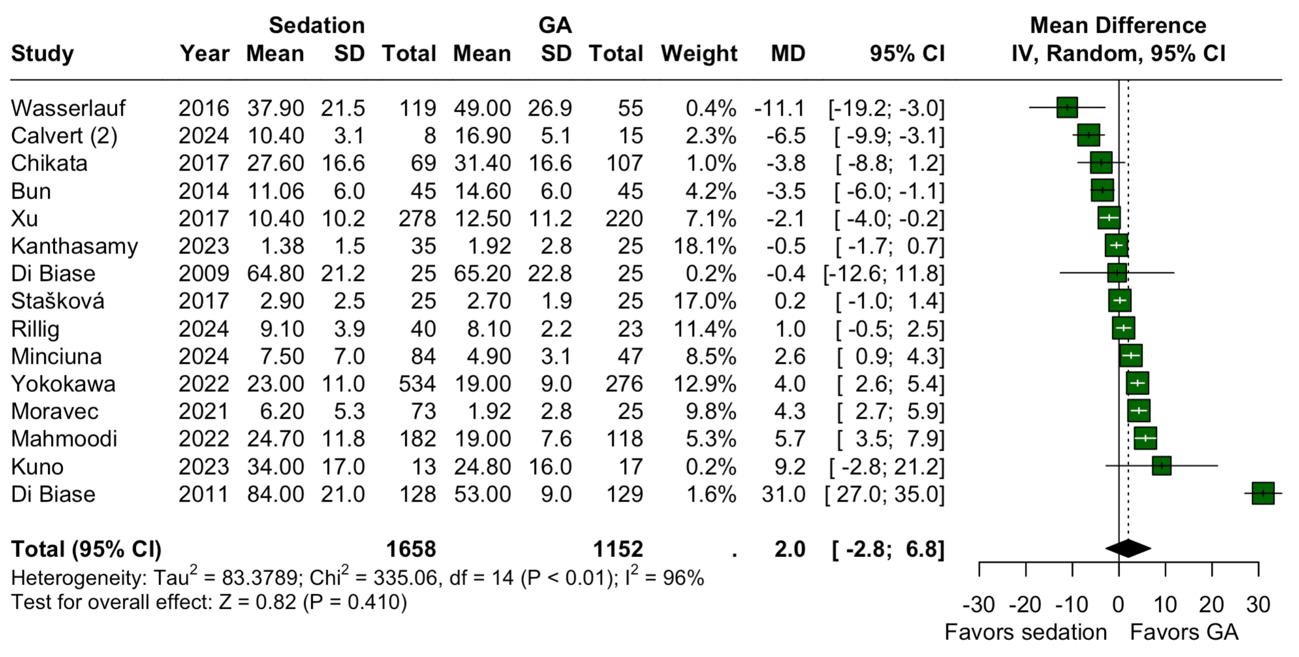
**

**Figure 6C.** Ablation time

**
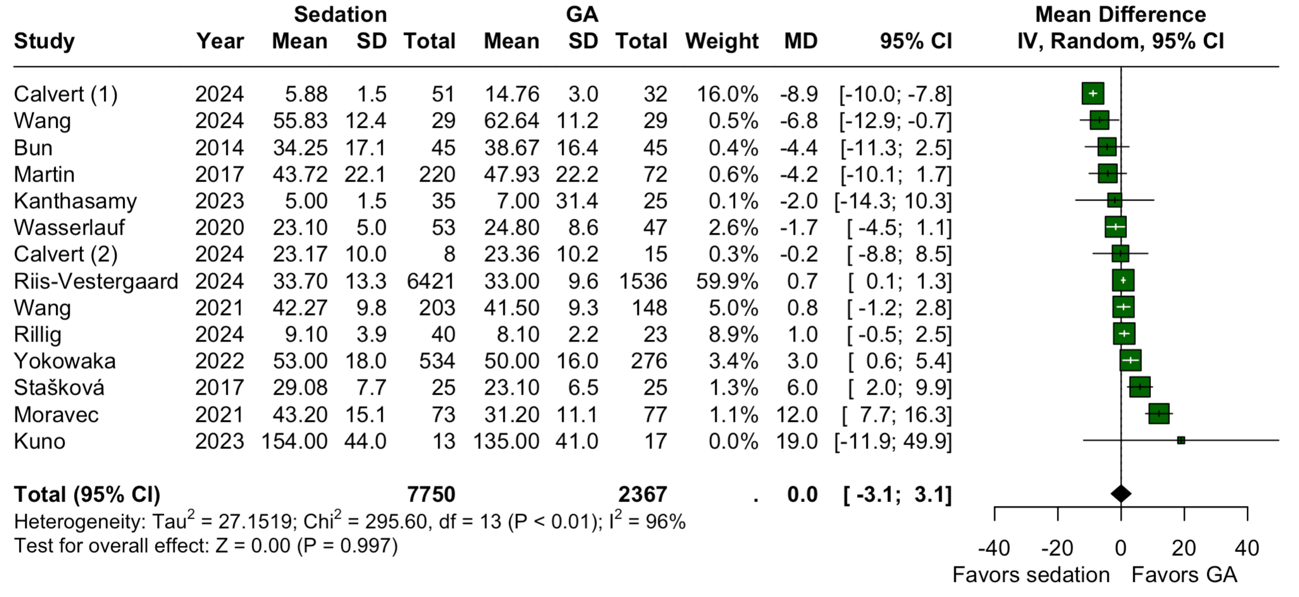
**

**Figure 6D.** Need for redo ablation

**
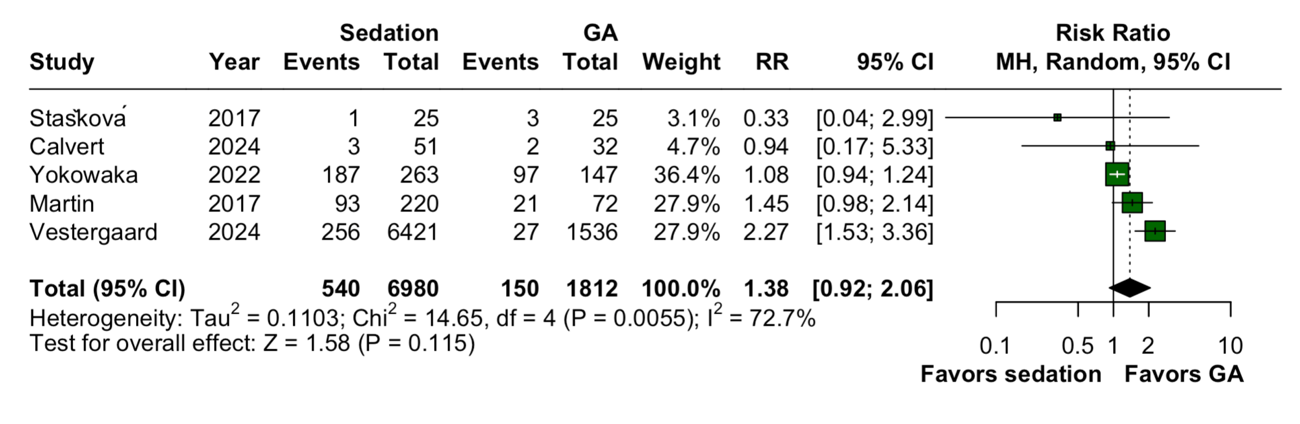
**

**Figure 6E.** Lab occupancy time


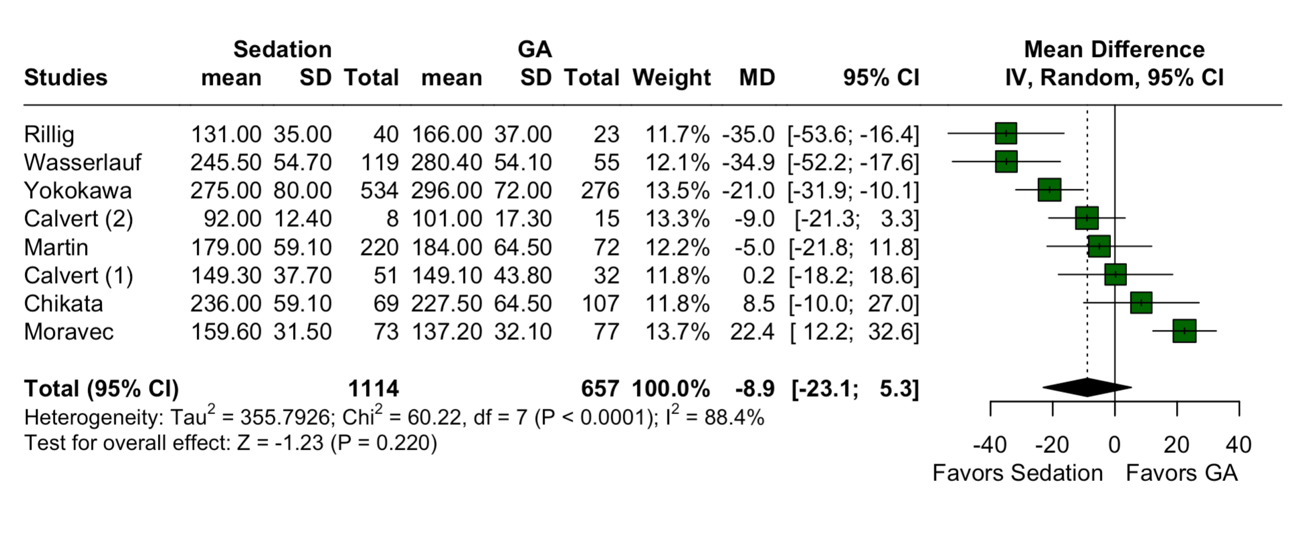


# Supplemental Figure 7. Risk of bias assessment of recurrence of atrial tachyarrhythmia

**Figure 7A.** ROBINS-I


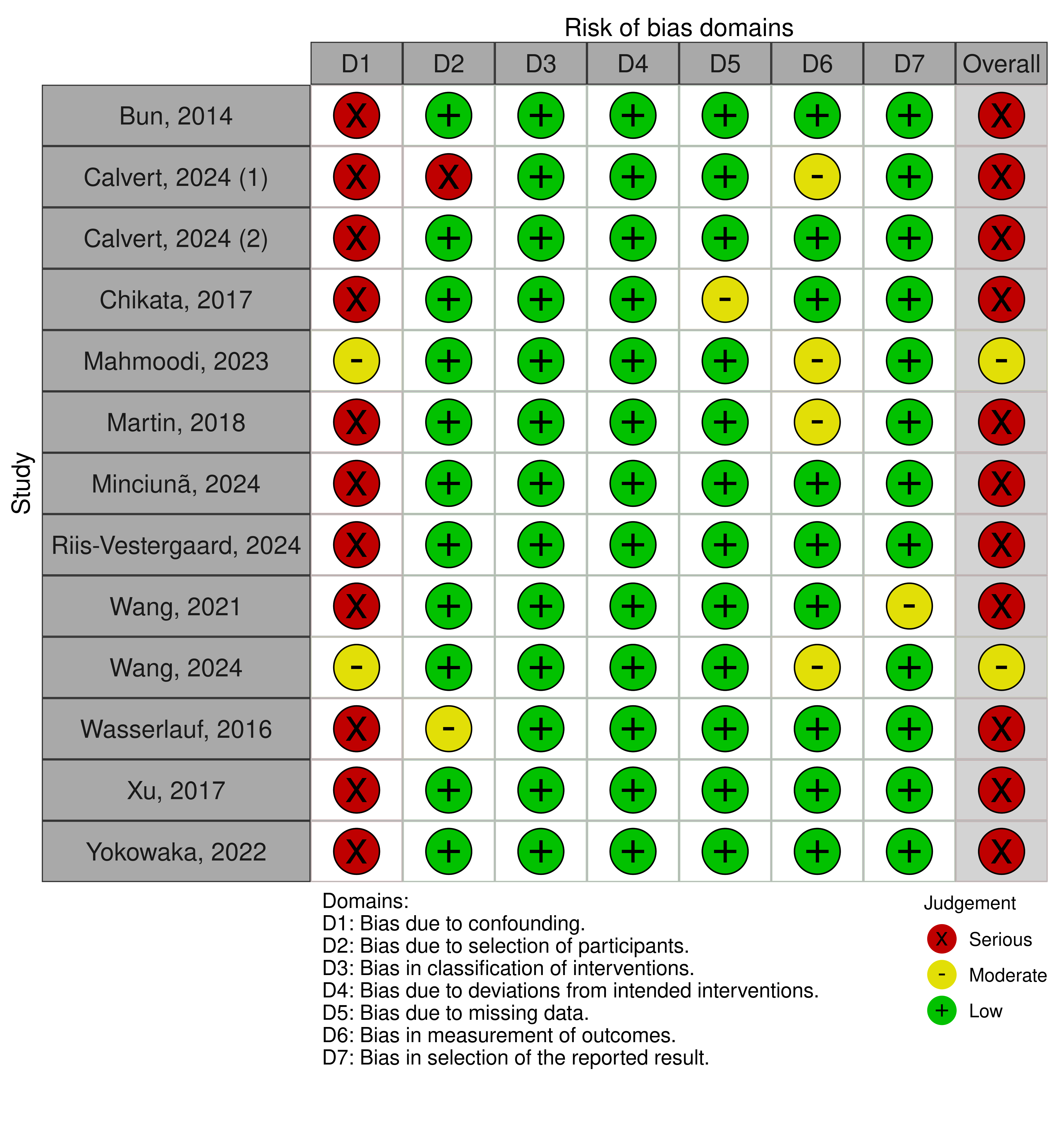


**Figure 7B.** RoB-2


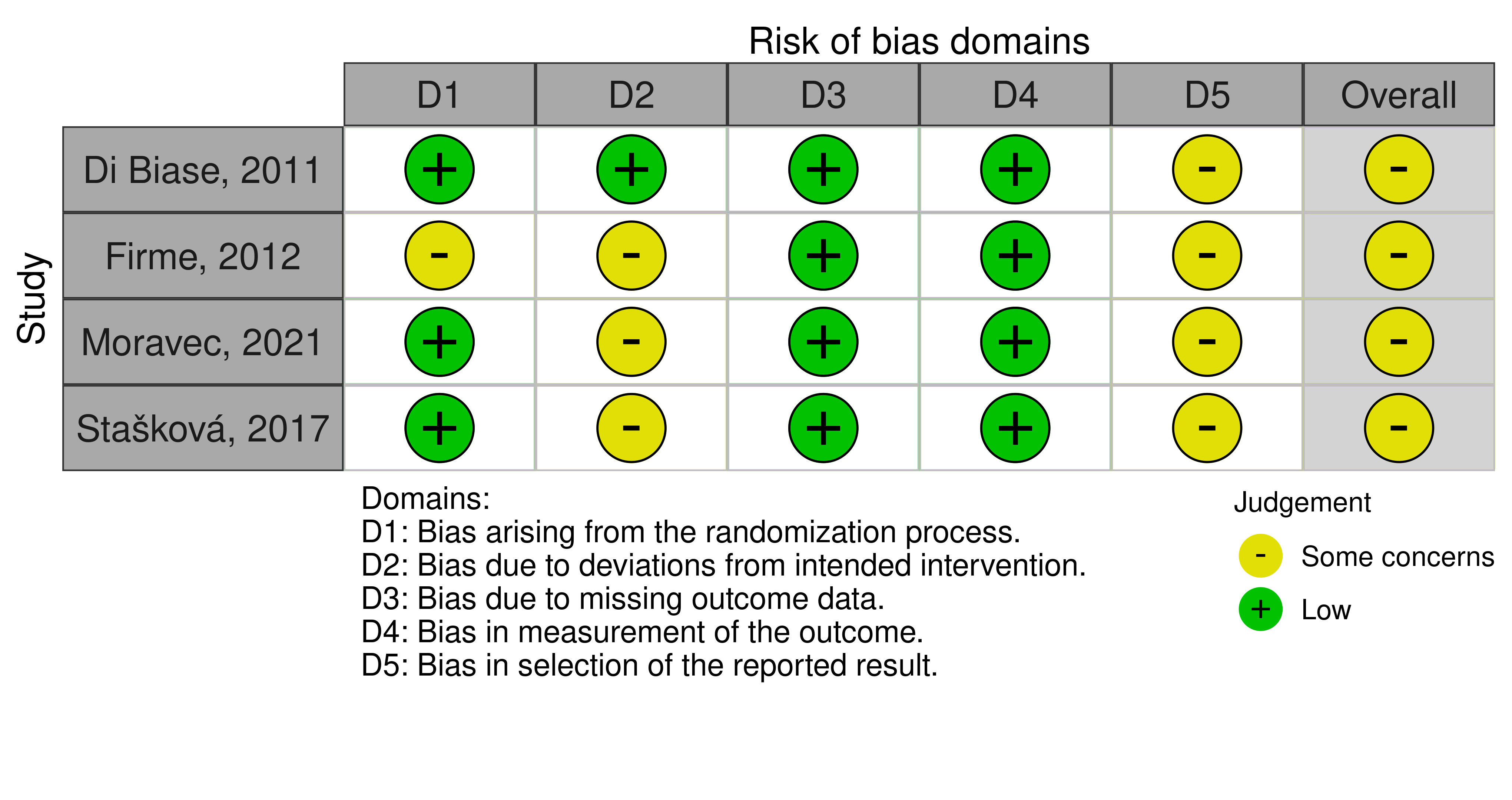


# Supplemental Figure 8. Funnel plot and Egger’s regression test for the primary endpoint


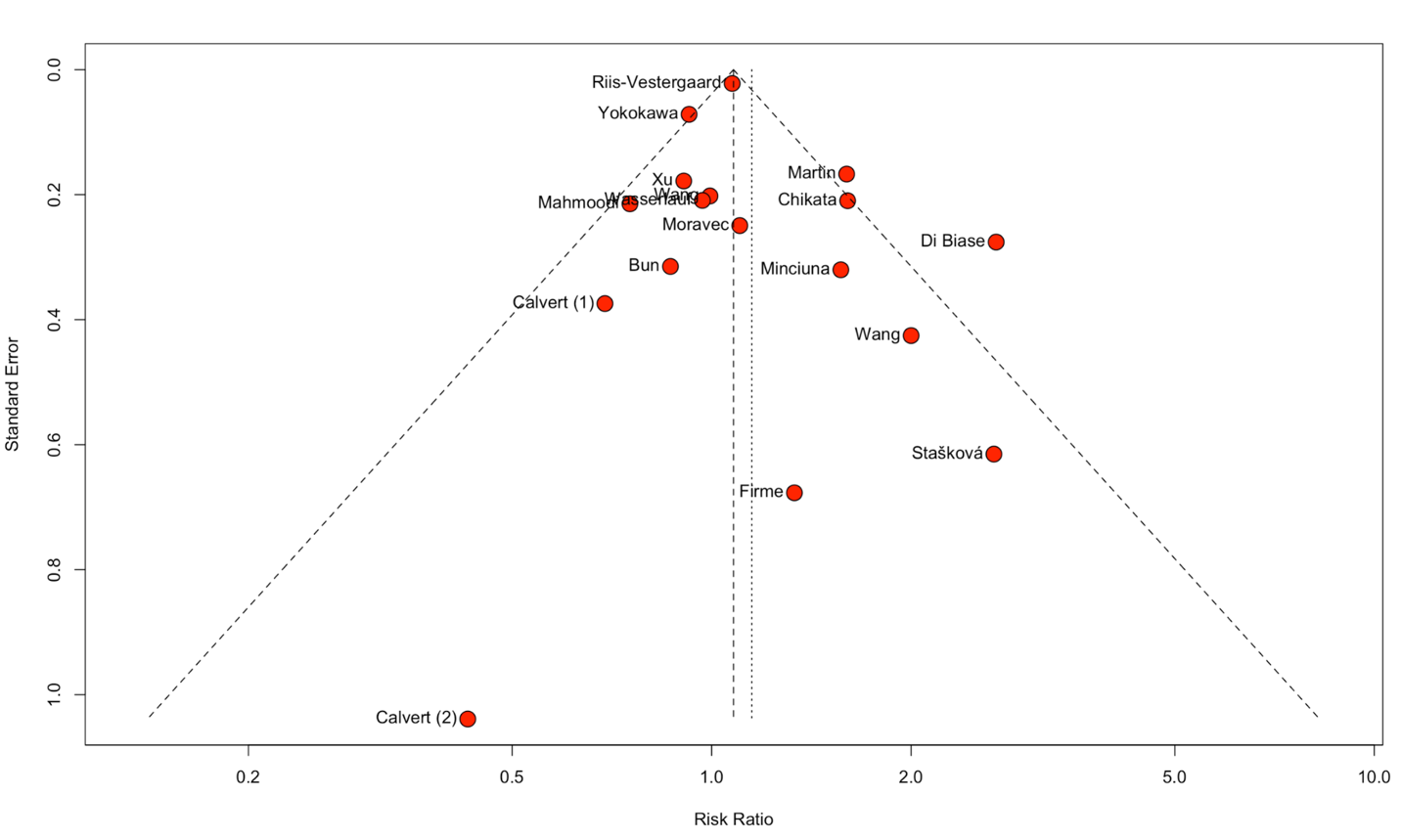


| Egger’s regression test | | | |
| --- | --- | --- | --- |
| Intercept | 95% CI | t | p-value |
| 0.351 | [-0.53] – [1.23] | 0.783 | 0.44 |
